# Anatomo-proteomic characterization of human basal ganglia: focus on striatum and globus pallidus

**DOI:** 10.1186/s13041-014-0083-9

**Published:** 2014-11-18

**Authors:** Joaquín Fernández-Irigoyen, María Victoria Zelaya, Teresa Tuñon, Enrique Santamaría

**Affiliations:** Clinical Neuroproteomics Group, Proteomics Unit, Proteored-ISCIII, Navarrabiomed, Fundación Miguel Servet, Irunlarrea Street, 31008 Pamplona, Spain; Neurological Tissue Bank, Navarrabiomed, Fundación Miguel Servet, 31008 Pamplona, Spain; Pathological Anatomy Department, Navarra Hospital Complex, Pamplona, Spain

**Keywords:** Basal ganglia, Striatum, Globus pallidus, Proteomics, Mass spectrometry, Bioinformatics

## Abstract

**Background:**

The basal ganglia (BG) are a complex network of subcortical nuclei involved in the coordination and integration of the motor activity. Although these independent anatomical structures are functionally related, the proteome present in each isolated nucleus remains largely unexplored. In order to analyse the BG proteome in a large-scale format, we used a multi-dimensional fractionation approach which combines isolation of anatomically-defined nuclei, and protein/peptide chromatographic fractionation strategies coupled to mass spectrometry.

**Results:**

Using this workflow, we have obtained a proteomic expression profile across striatum and globus pallidus structures among which 1681 proteins were located in caudate nucleus (CN), 1329 in putamen, 1419 in medial globus pallidus (GPi), and 1480 in lateral globus pallidus (GPe), establishing a BG reference proteome to a depth of 2979 unique proteins. Protein interactome mapping highlighted significant clustering of common proteins in striatal and pallidal structures, contributing to oxidative phosphorylation, protein degradation and neurotrophin signalling pathways. *In silico* analyses emphasized specific pathways represented in striatal and pallidal structures highlighting 5-hydroxytryptamine degradation, synaptic vesicle trafficking, and dopamine, metabotropic glutamate and muscarinic acetylcholine receptor pathways. Additional bioinformatic analyses also revealed that: i) nearly 4% of identified proteins have been previously associated to neurodegenerative syndromes, ii) 11% of protein set tends to localize to synaptic terminal, and iii) 20% of identified proteins were also localized in cerebrospinal fluid (CSF).

**Conclusions:**

Overall, the anatomo-proteomic profiling of BG complements the anatomical atlas of the human brain transcriptome, increasing our knowledge about the molecular basis of the BG and the etiology of the movement disorders.

**Electronic supplementary material:**

The online version of this article (doi:10.1186/s13041-014-0083-9) contains supplementary material, which is available to authorized users.

## Background

The basal ganglia (BG) and related nuclei consist of a variety of subcortical cell groups involved primarily in motor control, together with several roles in emotions and executive functions and behavior [1,2]. The term BG refers to nuclei embedded deep in the brain hemispheres (striatum or CN-putamen) and globus pallidus (GP), whereas related nuclei consist of structures located in the diencephalon (subthalamic nucleus), and mesencephalon (substantia nigra) [3]. The CN, the putamen, and the accumbens nucleus are all considered input nuclei, structures that receive incoming information from different sources such as cortex, thalamus, and substantia nigra (SN). The medial globus pallidus (GPi), and the SN pars reticulata (SNr) structures are the output nuclei, sending BG information to the thalamus. Finally, intrinsic nuclei such as the lateral globus pallidus (GPe), the subthalamic nucleus (STN) and the substantia nigra pars compacta (SNc) are located between the input and output nuclei [4].

Dopamine finely tunes striatal input as well as neuronal striatal activity, and modulates GPe, GPi, and STN activity. In the motor circuit, the striatum receives glutamatergic afferents from the cortex and thalamus and dopaminergic innervation from the SN. The putamen projects inhibitory GABAergic axons to both pallidal segments GPe and GPi which also receives glutamatergic fibers from the STN. The thalamus projects to the striatum and STN and the dopaminergic fibers also reach the GP, STN, thalamus, and brainstem [5].

Major cell types of BG neurons are highly specialized and are classified based on morphological, physiological and neurochemical properties. The striatum is the largest subcortical brain structure in the mammalian brain, containing projection neurons (medium-sized spiny neurons) and interneurons. Both types of cells are inhibitory neurons that use γ-aminobutyric acid (GABA) as the neurotransmitter [6]. Projection neurons could be also divided according to their projection targets, into those innervating the GPe nucleus and those projecting to the GPi and SNr. Both types of projection neurons differ also in the dopamine receptor subtype expressed and in the ability to inhibit/activate the adenyl-cyclase pathway [7]. At least, four types of GABAergic interneurons have been characterized according to their electrophysiological and neurochemical properties [8]. Both GP nuclei are composed of sparsely distributed GABAergic neurons with large somas, characterized by an enhaced expression of the calcium-binding protein parvalbumin [7].

In the last decade, different -omics strategies have been used to understand the molecular organization and complexity of different regions of the brain [9-12]. Specifically, quantitative proteomics based on a combination of 2-DE or ^18^O labelling with MS has been used to describe protein profiles of striatum in rat and macaque models of L-DOPA-induced dyskinesia [13-16] and also in several models of Huntington’s and Parkinson’s diseases [16-25]. In particular, proteomic technologies have been also applied in the analysis of the substantia nigra derived from parkinsonian subjects [26-28]. On the other hand, MS-based qualitative proteomics has been employed to profile the murine striatum proteome and secretome [29,30]. However, despite these efforts to identify and catalogue part of the murine proteins present in BG, only a very limited number of human proteins have been characterized in these independent and functionally related substructures that compose the BG.

Here we used anatomical, protein, and peptide fractionation strategies coupled to nanoLC-MS/MS to perform a shotgun proteome-wide analysis of the human globus pallidus (GPe and GPi), CN, and putamen proteomes in depth, and present a reference proteome map of the human BG. We report the identification of 2979 protein species in basal nuclei derived from 6 healthy patients. Using integrated *in-silico* studies, we provide an extensive overview of molecular functions based on gene ontology term enrichment, pathways studies, and interactome network analysis. This protein compilation present in human BG paves the way toward the complete molecular characterization of subcortical structures, and may be useful to understand the molecular basis of neurodegenerative movement disorders.

## Results

### Large-scale identification of human basal ganglia proteomes by mass spectrometry

**Figure 1:**
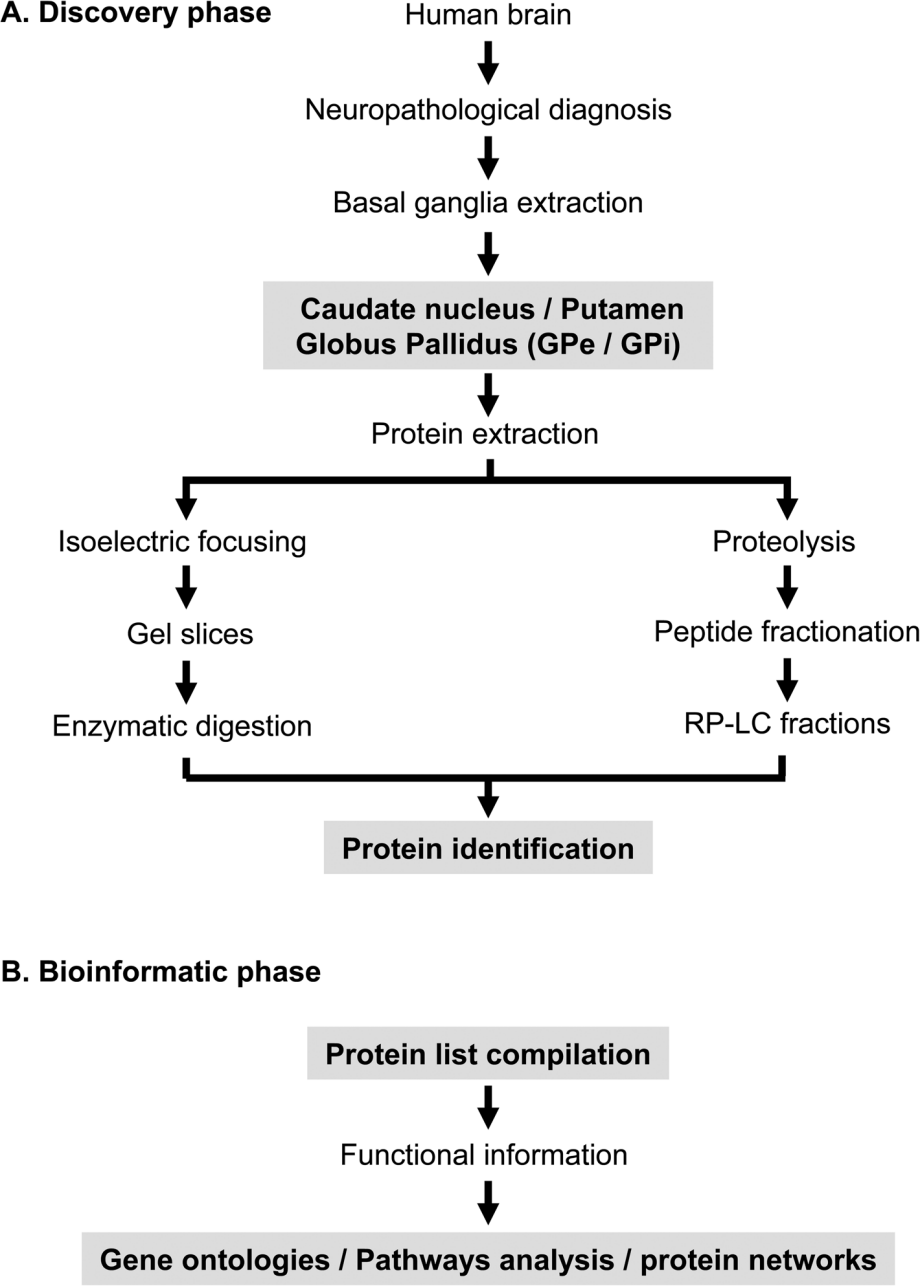
**An overview of the workflow used for identification of the BG proteomes.**

**Figure 2:**
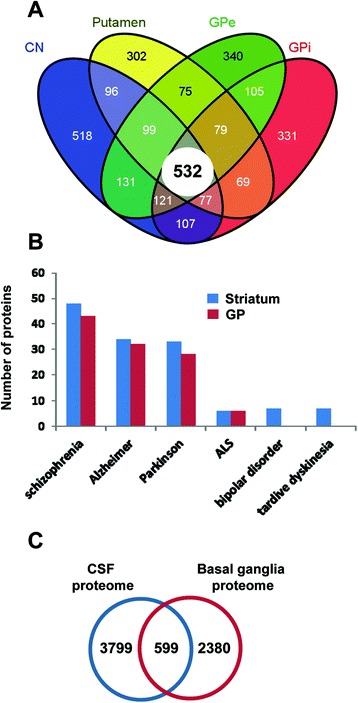
**Venn diagram of common and unique proteins between BG structures. A)** The distribution of common and distinct proteins, identified by 2 or more peptides, in CN, putamen, GPe, and GPi is shown. **B)** Graph showing the number of proteins associated with neurodegenerative syndromes according to Genetic Association Database. ALS, amyotrophic lateral sclerosis. **C)** Overlap of integrated BG proteome characterized in this study with CSF proteome datasets [32,33].

In the present study, we have used autopsy specimens of the right BG structures from six healthy human brains with the final goal to obtain a profound insight into the protein content and protein function of the nuclei that compose the BG. To reduce protein complexity, we used an integrated experimental workflow combining anatomical fractionation, protein IEF, and chromatographic-based methods coupled to mass spectrometry (Figure 1). For each basal nucleus, proteins were separated by IEF and the gel was sliced in 25 portions followed by in-gel trypsin digestion. The second approach involved in- solution digestion followed by off-line RP-LC at basic pH to separate the peptide mixture in 20 fractions. Replicate mass spectrometry measurements were performed in all peptide fractions for each basal nucleus. MS/MS data from each substructure were processed to identify peptides that gave rise to observed spectra, and proteins were inferred based on identified peptides. The number of non-redundant proteins identified with at least two unique peptides ranged from 1681 proteins for CN, 1329 for putamen, 1419 for GPi, and 1480 for GPe generating a BG reference proteome map of 2979 unique protein species, identified with a FDR lower than 1%. Complete lists of identifications and their corresponding scores are presented in Additional file 1: Table S1, Additional file 2: Table S2, Additional file 3: Table S3, Additional file 4: Table S4 and in ProteomeXchange repository (http://proteomecentral.proteomexchange.org). As shown in Figure 2A, 30% of the dataset (916 proteins) was exclusively detected in striatal structures (CN and putamen) while 26% (776 proteins) was identified only in pallidal nuclei (GPe and GPi). On the other hand, 532 proteins (18% of the protein set) overlapped between the four regions. According to Genetic Association Database [31], 100 striatal and 88 pallidal proteins have been previously linked to neurodegenerative syndromes such as schizophrenia, Parkinson’s disease, Alzheimer’s disease, and amyotrophic lateral sclerosis (Figure 2B and Additional file 5: Table S5). The integrated BG proteome dataset was also compared with previously published lists of human CSF proteome descriptions [32,33]. Interestingly, 599 proteins (20% of the protein set) were found to exist in both the BG and CSF proteomes (Figure 2C). A subset of these proteins is known to be involved in a plethora of central nervous system functions (Additional file 6: Table S6) and some of them are implicated in the development of neurological diseases such as Alzheimer’s, Parkinson’s, and Huntington’s diseases (Additional file 6: Table S6).

### Protein interactome map for common proteins detected in striatum and globus pallidus

**Figure 3:**
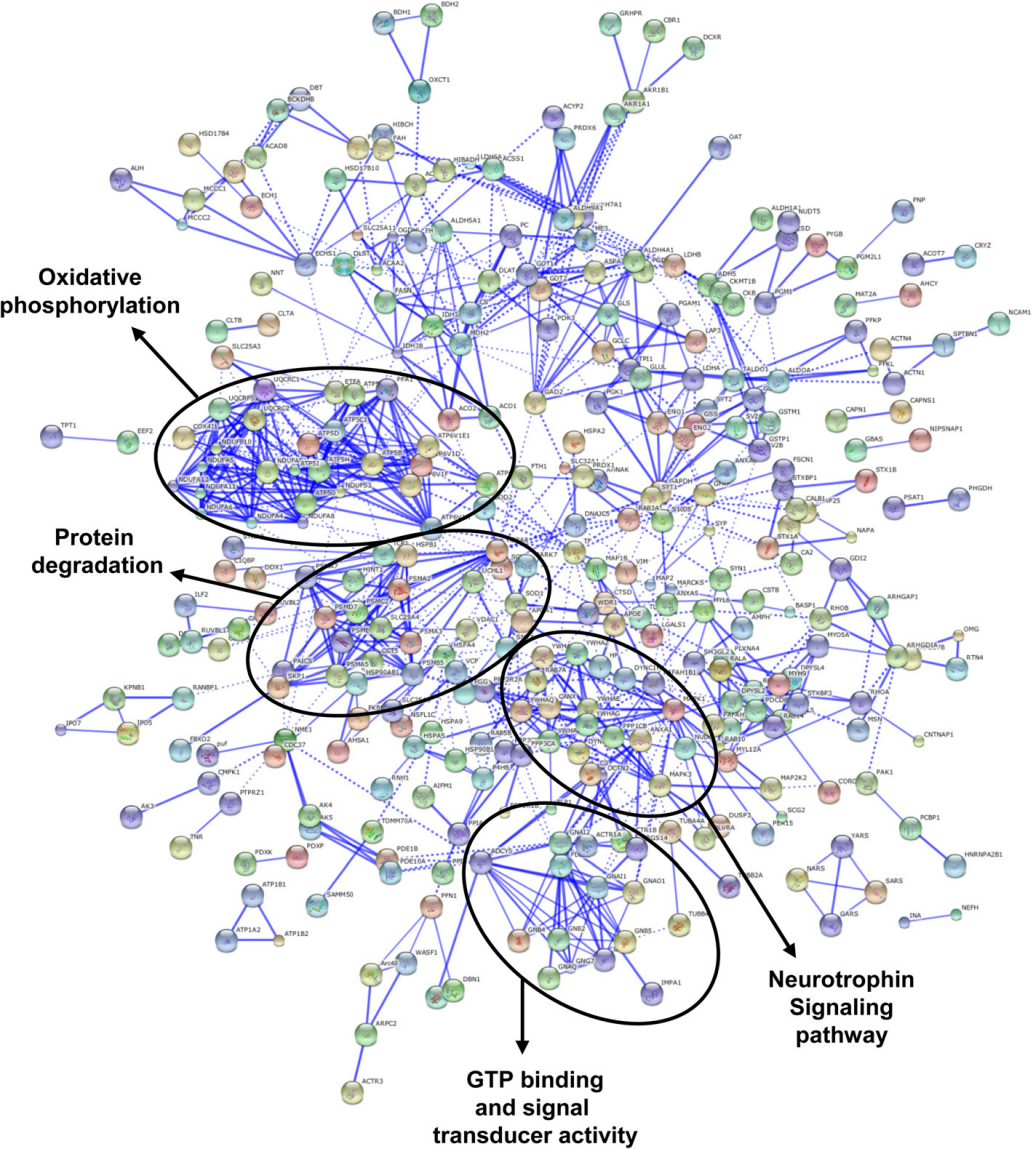
**Protein interactome network for shared proteome between pallidal and striatal structures.** Using the STRING software, proteins are represented with nodes and the interactions with continuous lines to represent direct interactions (physical), while indirect ones (functional) are presented by interrupted lines. Stronger associations are represented by thicker lines.

To enhance the analytical outcome of proteomic experiments, we have performed a proteome-scale interaction network merging the 532 common proteins detected in GPe, GPi, CN, and putamen using the STRING software [34]. This database includes interactions from published literature describing experimentally studied interactions, as well as those from genome analysis using several well-established methods based on domain fusion, phylogenetic profiling and gene neighbourhood concepts. Accordingly, a confidence score for every protein–protein association was assigned to the network. A higher score was assigned when an association is supported by several types of evidence. To minimize false positives as well as false negatives, all interactions tagged as “low-confidence” (<0.4) in STRING database have been eliminated from this study. Thus, the final network is composed by 335 nodes (proteins) and 941 edges (interactions) (Figure 3, Additional file 7: Figure S3 and Additional file 8: Table S7), establishing the first comprehensive interactomics map for the BG proteome. The topological analysis of this network demonstrated a central interplay with highly connected interactions and several sparsely connected sub-networks (Figure 3). The main central subset is composed by 27 nodes and is referred as the oxidative phosphorylation network (Figure 3). Other overlapping sub-networks are composed by interconnected nodes linked to GTP binding and neurotrophin signalling pathway and also by nodes involved in protein degradation (Figure 3).

### Contribution to the repertoire of human brain proteome

**Figure 4:**
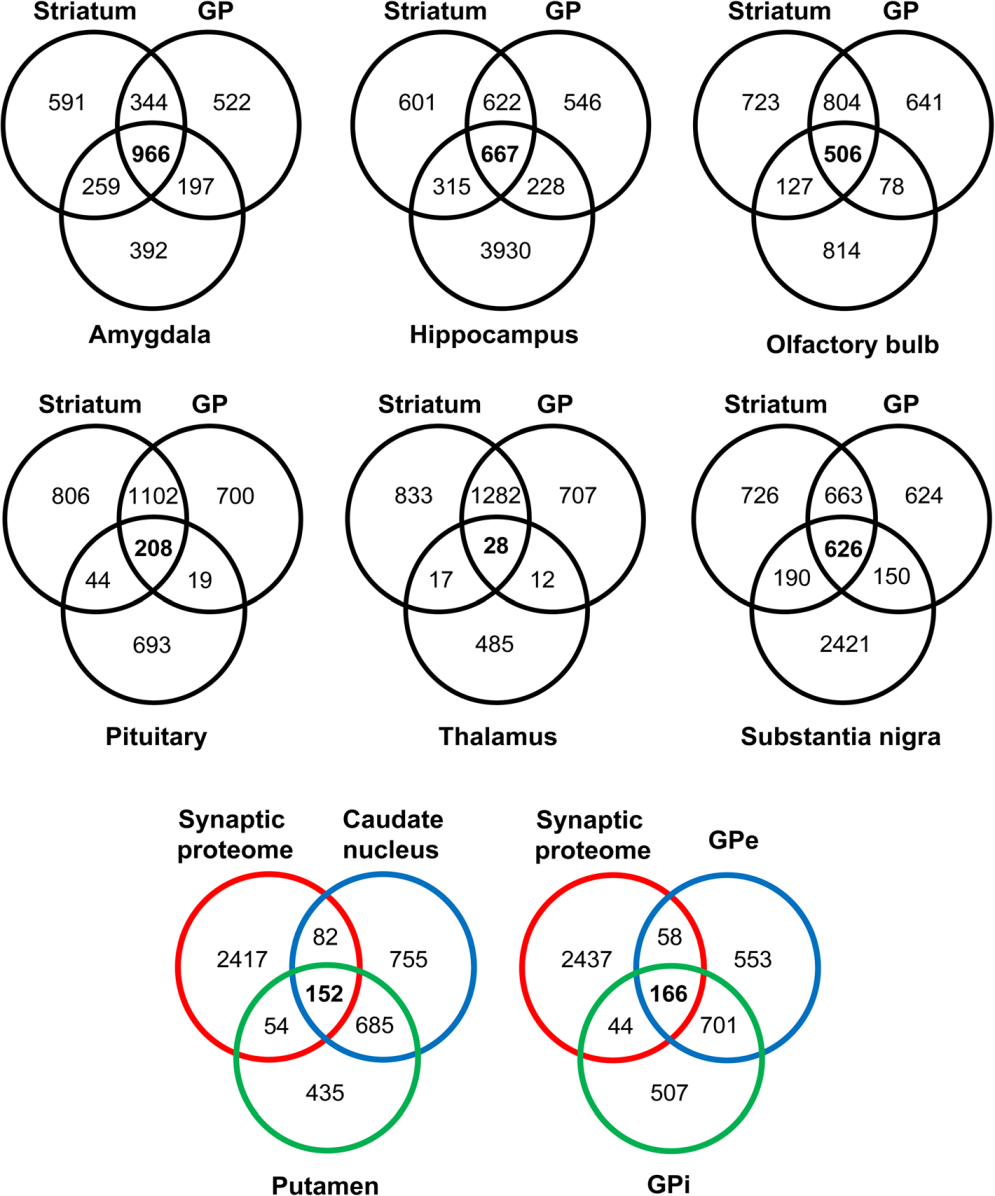
**Intersection of proteins derived from proteomic expression profiling of human limbic structures and synaptosomes with proteins identified in BG nuclei.** (*upper*) Protein expression data reported in amygdala [35], hippocampus [36], olfactory bulb [37], pituitary gland [38], thalamus [39], and sustantia nigra [26,28,40-42] have been considered. Striatum dataset contains proteins detected in CN and putamen while GP dataset encompasses proteins identified in the lateral and medial segments (GPe and GPi). Numbers represent the number of shared proteins in the respective overlapping areas. (*lower*) Venn diagrams representing the overlap between the “synaptic proteome” composed by the three major databases on synaptic proteins (C2G, SynsysNet, and Synaptome DB) [43-45] and the striatal and pallidal proteomes characterized in this study.

Protein identification data from the current study was compared with previous proteomic descriptions derived from large-scale proteomic studies of intimately related structures from human limbic system such as amygdala [35], hippocampus [36], olfactory bulb [37], pituitary gland [38], thalamus [39], and sustantia nigra [26,28,40-42] (Figure 4). On the other hand, combining the information stored in three repositories containing the largest number of synapse specific proteins (G2Cdb, Synaptome DB, and SynsysNet) [43-45], we have constructed an integrative synaptic proteome database containing 2705 synaptic protein-coding genes (Additional file 9: Table S8). Then, we have compared our protein compilation lists with the synaptic proteome database, detecting expression of 288 and 268 synaptic proteins in striatal and pallidal structures respectively (Figure 4 and Additional file 9: Table S8). In total, 346 proteins identified in BG (11% of the dataset) tend to localize to synaptic terminal.

### Functional metrics of human BG proteomes. Gene ontology analysis

To extract biological knowledge, the striatal and pallidal proteome datasets were functionally categorized based on Gene Ontology (GO) annotation code using DAVID software [46]. From striatal and pallidal datasets, 1393 and 1308 identifiers were considered for further analysis. With respect to the biological process and molecular function categories, an enrichment analysis has been performed against *Homo Sapiens* background using functional annotation clustering provided in DAVID tool. Respects to biological process ontology, 24 clusters are significantly enriched in BG nuclei respect to human genome (Additional file 10: Table S9). Some of the most significantly enriched biological processes included long-term strengthening of neuromuscular junction, aerobic respiration, regulation of ubiquitin-protein ligase activity, and glucose and ATP catabolism. Representative biological process categories from each cluster in striatal and pallidal structures are shown in Additional file 7: Figure S4.

Respects to molecular function ontology, 10 clusters are significantly enriched in the BG proteome dataset (Additional file 11: Table S10). Intramolecular oxidoreductase, aminoacyl-tRNA ligase, hydrogen ion transmembrane transporter, and ATPase activities, were the most significantly enriched molecular functions in our dataset. Representative molecular function categories from each cluster in striatum and globus pallidus are represented in Additional file 7: Figure S5. Respect to cellular component ontology, a significant proportion of striatal and pallidal proteins consisted of cytosolic, mitochondrial, cytoskeletal proteins together with plasma membrane and vesicle proteins (Additional file 7: Figure S6A and Additional file 11: Table S10). Additionally, a neuron-specific cell component analysis was also performed, detecting proteins associated to neuron projection, synapse, axon, and dendrites between others (Additional file 7: Figure S6B and Additional file 11: Table S10).

### Functional metrics of human BG proteomes. Pathways analysis

**Figure 5:**
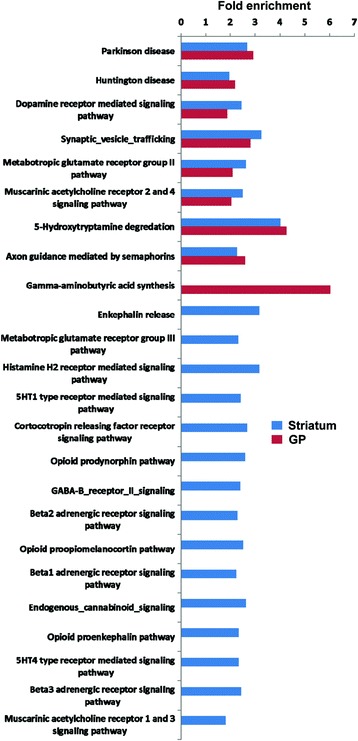
**Specific-Neuronal Pathway analysis for the BG proteomic expression profile.** Protein clusters significantly enriched in striatal and pallidal proteome datasets (fold enrichment >1.5, EASE p-value <0.01) are shown. Fold enrichment refers to the number of relevant striatal or pallidal protein species represented in each cluster relative to random expression of all genes in the human genome. A complete characterization of each cluster is shown in Additional file 13: Table S12.

A complementary analysis of biological processes was performed with a search of KEGG pathways [47], that are over-represented in human BG nuclei. The 35 pathways represented with a high statistical significance (fold enrichment >1.5; EASE p-value <0.01) are shown in Additional file 12: Table S11. Four of these pathways are clearly related to neurological disorders such as Parkinson’s, Alzheimer’s, Huntington’s, and Prion diseases. Among the KEGG pathways, oxidative phosphorylation, proteasome, and neurotrophin signalling pathway, are of particular interest because they partially reinforce the BG interactome map. Strong representation of carbohydrate, lipid, and amino acid metabolism, together with the regulation of neurological system process such as lon-term potentiation/depression and endocytosis, parallels with previous observations that GO Terms “glucose metabolism/catabolism”, aminoacyl-tRNA ligase activity”, “Fatty acid beta oxidation”, “regulation of synaptic transmission” and “neurotransmitter transport” are highly enriched in basal nuclei. In order to gain a more detailed description of the molecular mechanisms represented in striatum and globus pallidus, subsequent analyses were performed to explore the striatal and pallidal proteome distributions across specific reactions using the PANTHER classification system [48]. Interestingly, as shown in Figure 5, some statistically over-represented processes in both structures were directly relevant to neurotransmitter release, electrical machinery and synaptic plasticity (Additional file 13: Table S12). Specifically, we found a significant enriched pathway focused on gamma-aminobutyric acid (GABA) synthesis in pallidal dataset while in the case of striatum, most of enriched pathways are related to beta-adrenergic receptors and endogenous opioid system. The observation that there are protein clusters differentially enriched between striatal and pallidal structures related to specific biological process and molecular function ontologies is not relevant since all protein clusters were represented in both structures but their statistics did not reach the minimum statistical requirements.

### BG proteomics as a complement of the human brain transcriptome

**Figure 6:**
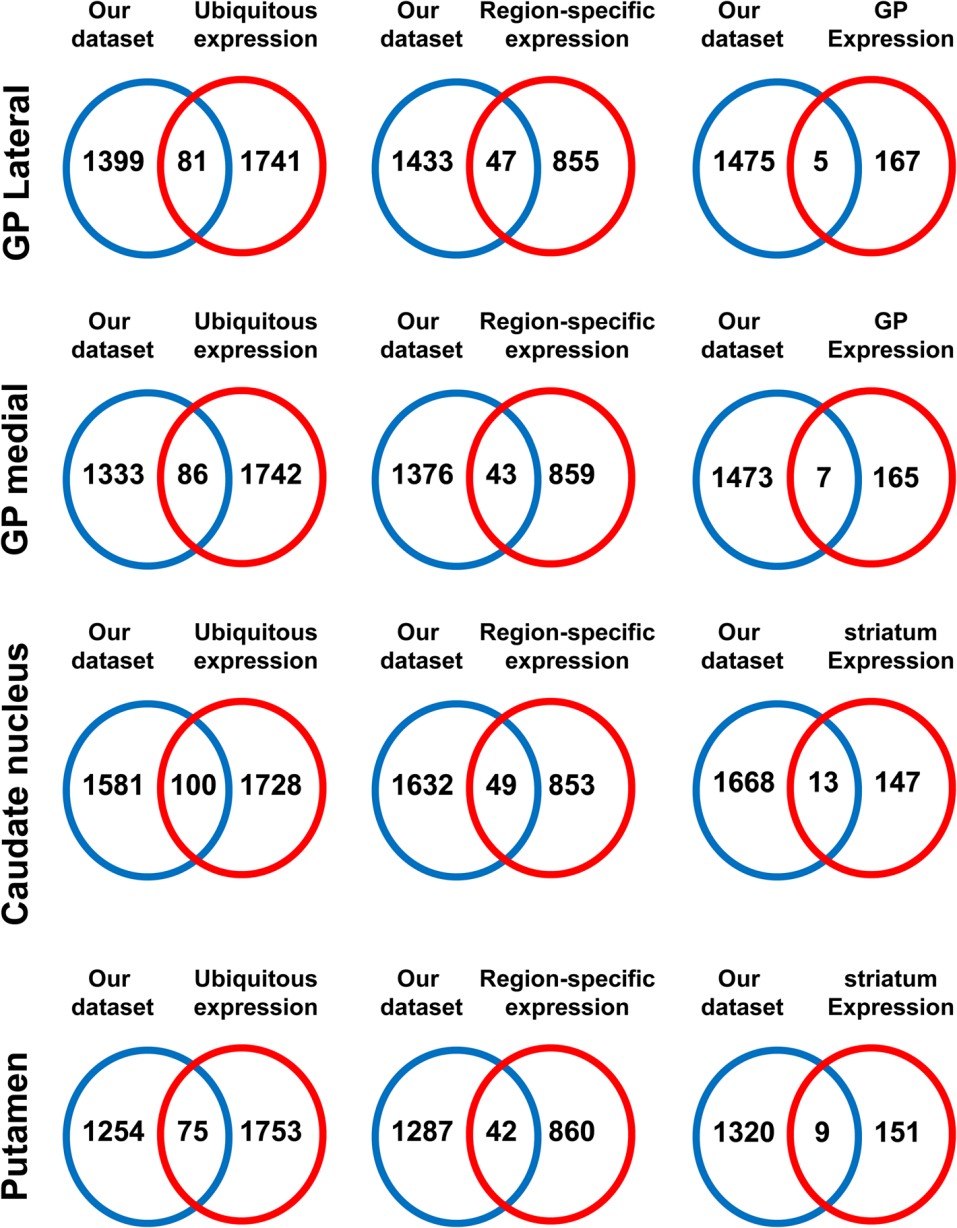
**Anatomical correlation between pallidal and striatal proteomic expression profiling and human brain transcriptome.** Protein datasets derived from BG structures were intersected with transcriptomic expression profiling obtained in human brain [11]. In this case, we have considered genes with brain-wide expression (ubiquitous expression) or within local ubiquity (region-specific expression) in neocortex, myelencephalon, hippocampal formation, dorsal thalamus, striatum, mesencephalon, cerebellar cortex, pontine tegmentum, cingulate gyrus, basal pons, claustrum, and globus pallidus [11]. See Additional file 14: Table S13 for more details.

We have correlated our proteomic fingerprints with the anatomical map of the human brain transcriptome stored in the Allen Brain Atlas [11] in order to analyze the interrelationship of expressed genes and proteins at different levels of organization. First, we have interlocked each BG proteomic expression profiling with transcriptomic data from genes with brain-wide (global) or within structure (local) ubiquity (Figure 6 and Additional file 14: Table S13). Second, transcriptome expression data from genes specifically expressed in striatum and globus pallidus [11] was checked against the anatomo-proteomic expression data derived from BG structures (Figure 6 and Additional file 14: Table S13). As shown in Figure 6, we have detected pallidal protein expression for 81 and 86 genes (in GPe and GPi respectively) and striatal protein expression for 100 and 75 genes (in CN and putamen respectively) with ubiquitous expression across human brain (Additional file 14: Table S13). In contrast, proteomic screens have revealed pallidal protein expression for nearly 40 genes with region-specific expression, whereas in the case of striatum, we have obtained protein expression data for 49 and 42 region-specific genes in CN and putamen respectively (30 genes common in both structures) (Figure 6 and Additional file 14: Table S13). From 172 region-specific genes that express in globus pallidus, we have obtained protein evidence for 5 and 7 genes in GPe and GPi respectively (4 common proteins in both structures) whereas 13 out of 160 region-specific genes that express in striatum have been detected at protein level in CN (9 genes in the case of putamen) (Figure 6 and Additional file 14: Table S13).

### Specific protein products of major cell type markers in BG datasets

Additional analyses were performed to detect cell type-specific information in BG by detecting protein evidence for well-known transcriptomic markers of major cell classes [49,50]. Protein expression of marker genes of oligodendrocytes (*CNP*, *HSPA2*, *MBP*, *MOG*, *PLP1*), astrocytes (*ATP1A2*, *GFAP*, *GLUL*, *HAPLN1*, *SLC4A4*, *AHCYL1*), and neurons (*PGM2L1*, *GDA*, *MAP2*, *MAP1B*, *SCG2*, *SNAP25*) was detected in all four structures (Table 1). In addition, protein products of other oligodendrocyte marker genes such as *BCAS1*, *ENPP6*, and *PPP1R14A* were found in pallidal structures. Protein expression was also detected for additional astrocyte markers such as *SLC1A2* (in GPe, GPi, and putamen), *ALDOC* (in GPi, CN, and putamen), *METTL7A* (in GPi and putamen), and ALDH1L1 in CN. Respect to neuronal markers, protein products for *PLCXD3*, SLC12A5, NRCAM, and NRXN1 genes were also identified across striatal and/or pallidal structures (Table 1).

**Table 1:** **Detection of specific protein products of major cell type markers in basal nuclei by proteomics**

**Markers**	**Protein products**	**Code**	**Gpe**	**Gpi**	**CN**	**Put.**
**OLs**						
*CNP*	2′,3′-cyclic-nucleotide 3′-phosphodiesterase	P09543	++	++	++	++
*HSPA2*	Heat shock-related 70 kDa protein 2	P54652	++	++	++	++
*MOG*	Myelin oligodendrocyte glycoprotein	Q5STM1	++	++	++	++
*PLP1*	Proteolipid protein 1	A8K9L3	++	++	++	++
*MBP*	Isoform 3 of Myelin basic protein	P02686-3	++	---	++	++
	Myelin basic protein	P02686	---	++	---	---
*BCAS1*	Isoform 2 of Breast carcinoma-amplified sequence 1	O75363-2	++	---	---	---
*ENPP6*	Ectonucleotide pyrophosphatase/phosphodiesterase member 6	Q6UWR7	---	++	---	---
*PPP1R14A*	Protein phosphatase 1 regulatory subunit 14A	Q96A00	---	++	---	---
**Astrocytes**						
*ATP1A2*	Sodium/potassium-transporting ATPase subunit alpha-2	B1AKY9	++	++	++	++
*GFAP*	Glial fibrillary acidic protein	P14136	++	++	++	++
*GLUL*	Glutamate-Ammonia Ligase	A8YXX4	++	++	++	++
*HAPLN1*	Hyaluronan and proteoglycan link protein 1	P10915	++	++	++	++
*AHCYL1*	Putative adenosylhomocysteinase 2	O43865	++	++	++	++
*ALDOC*	Fructose-bisphosphate aldolase C	P09972	---	++	++	++
*SLC4A4*	Solute carrier family 4 Na(+)/HCO3(-) cotransporter 4 isoform 2	Q9Y6R1-2	++	---	++	---
	Solute carrier family 4 Na(+)/HCO3(-) cotransporter 4	Q9Y6R1	---	---	---	++
	Solute carrier family 4 Na(+)/HCO3(-) cotransporter 4 variant	A5JJ20	---	++	---	---
*SLC1A2*	Excitatory amino acid transporter 2	P43004	++	++	---	---
	Solute carrier family 1, member 2	A2A2U1	---	---	---	++
*METTL7A*	Methyltransferase-like protein 7A	Q9H8H3	---	++	---	++
*ALDH1L1*	Cytosolic 10-formyltetrahydrofolate dehydrogenase	O75891	---	---	++	---
**Neurons**						
*PGM2L1*	Glucose 1,6-bisphosphate synthase	Q6PCE3	++	++	++	++
*GDA*	Guanine deaminase	Q9Y2T3	++	++	---	++
	Guanine deaminase isoform 3	Q9Y2T3-3	---	---	++	---
*MAP2*	Isoform 3 of Microtubule-associated protein 2	P11137-3	++	++	++	++
	MAP2 protein (Fragment)	Q6NYC5	++	++	++	++
*MAP1B*	Microtubule-associated protein 1B	P46821	++	++	++	++
*SCG2*	Secretogranin-2	P13521	++	++	++	++
*SNAP25*	Synaptosomal-associated protein 25	P60880	++	++	++	++
*SLC12A5*	Solute carrier family 12 member 5	Q9H2X9	++	---	---	---
	cDNA FLJ75342, similar to solute carrier family 12 member 5	A8K143	---	++	++	++
	Isoform SNAP-25a of Synaptosomal-associated protein 25	P60880-2	---	---	++	++
*PLCXD3*	similar to PI-specific phospholipase C, X domain containing 3	B3KXD1	++	---	++	++
*NRCAM*	Isoform 2 of Neuronal cell adhesion molecule	Q92823-2	---	++	---	---
	Neuronal cell adhesion molecule	Q92823	---	---	---	++
*NRXN1*	Neurexin-1-alpha	Q9ULB1	---	---	++	++

(++) indicates protein evidence and (---) non-detected. Code refers to the UniProtKB/Swiss-Prot entry for each protein. *OLs*, Oligodendrocytes.

## Discussion

The complex structural and functional organization of the brain is reflected by the selective spatial and temporal expression of its resident proteins [10]. Given its segmented functions, characterization of protein profiles within specific regions of the adult brain forms an essential part of unearthing the molecular basis for structure specialization and perturbation associated with neurological disorders.

In this work, we have performed an anatomically comprehensive proteomic mapping of human BG in order to gain new insight into the protein content and protein function of striatal and pallidal nuclei. Using complementary protein and peptide fractionation methods coupled to tandem mass spectrometry, we have identified 2979 unique proteins in BG corresponding to more than 5900 protein expression features distributed across CN, putamen, GPe, and GPi.

Although striatal and pallidal structures are composed by different specialized types of neurons with highly distinct transcriptomic profiles [6,7,11], more than 500 proteins have been commonly detected in CN, putamen, GPe, and GPi structures. This panel of proteins takes part in a global protein interactome map, indicating that oxidative phosphorylation, protein degradation, and neurotrophin signalling are active pathways mediated by overlapping protein interactors in striatal and pallidal structures.

Although our study has uncovered many intricacies in protein expression in BG, there are potential limitations of our study that warrant discussion. We analysed dissected tissues that contained multiple cell types, thus diluting the proteomic contribution of any one specific cell type. Furthermore, the number of structures analysed so far is not sufficient to investigate the full magnitude of proteomic expression in BG. Moreover, we have employed pooled samples for each independent substructure. Although all individuals were males of similar age and ethnicity, our data do not capture population or sex diversity. Taking into account that human brain transcriptome is highly dynamic during neurodevelopment [10,11], additional proteomic studies employing post-mortem brains of clinically unremarkable donors representing males and females with different ages and ethnic backgrounds are necessary to estimate the consistency of the proteomic profile obtained in this study.

One of the goals of the present study was to generate extensive and robust data on the functional groups of proteins present in human BG. To identify biologically relevant pathways from large-scale BG proteome data, we have undertaken a system biology approach performing different molecular network and pathways analysis using gene ontologies, KEGG pathways, and PANTHER classification system [46-48]. Although each bioinformatics platform produced diverse results, they commonly point out that tricarboxylic acid cycle, ubiquitin proteasome pathway and energy metabolism are the general over-represented processes in basal nuclei as might be expected given the high metabolic demands of neurons.

In agreement with different brain proteomic studies [32,35,37], a high proportion of identified proteins present catalytic and binding activities. We have not detected a clearly distinguishable pattern of molecular categories between striatal and pallidal structures. Several reasons may explain the functional parallelism observed in both structures. First, our workflow is based on analysing un-fractionated tissue homogenates using a Q-TRAP instrument [51] without employing previous sequential extraction of proteins (for example enriched organelle fractions, synaptosomes, etc.). This implies that our proteomic datasets may correspond to highly abundant proteins and housekeeping enzymes and tend to represent the majority of proteins identified in unfractionated neural tissue, hampering the identification of less abundant proteins with functional relevance. Future studies employing high-resolution instruments will increase the quality of BG proteome data in terms of high resolving power, mass accuracy, and high sequencing speed, generating novel proteomic data with high impact from a functional point of view [52,53]. Second, a high proportion (30%) of pallidal and striatal proteomes encompasses uncharacterized, putative or predicted proteins based on genomic sequence data, limiting the extraction of functional information from bioinformatics resources. This is in accordance with data obtained in human cortex, thalamus, and amygdala where also more than 20% of the proteins identified by shotgun proteomic methods could not be assigned to biological processes [32,35,39,54]. However, we have obtained a cell type-specific information in basal nuclei identifying protein products for well-known markers of major cell classes derived from large-scale transcriptomic studies [49,50] indicating that shotgun proteomic approaches may be useful to identify specific protein isoforms of cell markers, obtaining complementary information that adds value to the conventional detection by immunohistochemical staining.

We wish to emphasize that even though certain proteins were identified in a particular BG structure, this does not necessarily imply their absence in other nuclei (see Additional file 15: Figure S7). Our data indicate that a considerable proportion of this protein list was identified with a low number of peptides suggesting that the discovery of this subset of proteins in a specific region may be due to technical bias rather than specific protein enrichment with respect to other structures.

We have obtained a deep functional analysis of the striatum and globus pallidus, detecting a plethora of neuronal molecular events enriched in basal nuclei. In agreement with high throughput proteomic analyses of other brain regions [32,37], enriched functional categories focused on carbohydrate, lipid, and amino acid metabolism were also detected in striatum and globus pallidus. In addition, we have found a considerable proportion of proteins (11% of the dataset) that tends to localize to synaptic terminal suggesting a potential role in the regulation of synaptic transmission and neurotransmitter transport. For example, functional analyses allowed us to classify different set of proteins in specific-neuronal pathways across pallidal and striatal structures such as serotonin degradation (8 proteins), dopamine receptor (30 proteins), metabotropic glutamate receptor (18 proteins), muscarinic acetylcholine receptor (22 proteins), and endogenous opioid system (10 proteins).

In order to expand our knowledge about the global system biology of human brain, we interlocked regional transcriptomic and proteomic signatures by focusing our analyses on genes with global or local ubiquity [11]. Our anatomo- proteomic analysis partially complements the human brain transcriptome since we have obtained protein expression data for an appreciable number of genes with proven transcriptomic evidence in basal nuclei and other structures across the human brain. Specifically, qualitative proteomics allow us to detected protein expression (in GPe and GPi structures) for 8 genes (Thiomorpholine-carboxylate dehydrogenase, RuvB-like 2, Calcium/calmodulin-dependent 3′,5′-cyclic nucleotide phosphodiesterase 1B, Hyaluronan and proteoglycan link protein 1, Neuronal pentraxin-1, Synaptic vesicle glycoprotein 2B, Ephrin type-A receptor 6 and CaM kinase-like vesicle-associated protein) that tend to be differentially regulated between both pallidal structures [11].

On the other hand, *in silico* analysis points out that nearly 4% of identified proteins in BG have been previously associated to neurodegenerative syndromes, and 20% of identified proteins were also localized in cerebrospinal fluid (CSF) from healthy subjects [33,55]. From the point of view for biomarker discovery, it is crucial to identify the presence of brain tissue analytes in CSF, a readily accessible resource for biomarker development pipelines. Therefore, this comprehensive BG protein dataset may serve as a reference list of human BG proteome for biomarker research of movement disorders and other neurodegenerative diseases, being a useful resource to establish quantitative targeted analysis of potential BG protein biomarkers by MRM (Multiple Reaction Monitoring) assays [56,57].

## Conclusions

We have carried out, to the best of our knowledge, the first comprehensive analysis of the proteome present in human caudate nucleus, putamen, GPe, and GPi. Our results provide a broad functional analysis of 2979 non-redundant striatal and pallidal proteins, being the first step toward the complete characterization of the tangled molecular architecture that composes de BG. This molecular fingerprint together with previous proteomic profiling of specialized brain regions [32,35-39,54,58-61] contribute to the repertoire of the human brain proteome, providing fundamental information for the recently officially launched Human Proteome Project and the BRAIN Initiative [62,63]. Taking into account that the central nervous system poses many specific challenges in terms of proteomics, given the large number of different neuronal cell types that are intermixed and that exhibit distinct patterns of gene and protein expression [10,11,64], the development of specific isolation protocols of single-cell types together with novel developments in shotgun proteomic strategies [65] will allow to explore the proteome profiling of each subcortical nucleus individually, boosting the molecular knowledge of the BG.

## Methods

### Sample collection

According to the Spanish Law 14/2007 of Biomedical Research, inform written consent form of the Neurological Tissue Bank of Navarra Health Service was obtained for research purposes from relatives of patients included in this study. The 6 patients were male and ages ranged from 29 to 61 years, with no known neuropsychiatric or neurological history (control cases) (Additional file 7: Figure S1). According to standard practices in place at the neurological tissue banks, the left cerebral hemisphere was progressively frozen and stored at −80°C (*post-mortem*-interval: 6-15 h). The diagnosis was carried out on the left cerebral hemisphere. Therefore, the BG assessed in this study were the right ones. Following fixation in 10% formaldehyde for approximately three weeks, the brains were sectioned according to the recommendation guide proposed by BrainNet Europe [66]. After a macroscopic study, immunohistochemistry analysis was performed in different brain regions using specific antibodies against Tau protein, β amyloid, TDP-43, PrP, α-synuclein, ubiquitin and α- β crystalline. These brains did not show significant pathology and were considered to be healthy. In particular, the immunohistochemical study of the BG showed normal tissue without appreciable abnormalities. The recognition of basal ganglia structure by the neuropathologists was as follows: the caudate nucleus (CN) is a C-shaped structure that is closely associated with the lateral wall of the lateral ventricle. The putamen is also a large structure that is separated from the CN by the anterior limb of the internal capsule. The globus pallidus (GP) is divided into two segments: the internal (or medial) segment and the external (or lateral) segment. Both are separated by the medial medullary lamina. The GP is separated from the putamen by a thin layer of white matter called the lateral medullary lamina. A representative image of BG is shown in Additional file 7: Figure S2.

### Sample preparation for proteomic analysis

BG specimens were obtained from frozen brain sections using sterile biopsy punches (size: 3–4 mm) and homogenized in lysis buffer containing 7 M urea, 2 M thiourea, 4% (w/v) CHAPS, 50 mM DTT. The homogenates were spinned down at 100.000 × g for 1 h at 15°C. Protein concentration was measured in the supernatants with the Bradford assay kit (Biorad). Prior to proteomic analysis, the individual BG samples were grouped into 4 independent pools (GPe, GPi, putamen, and CN) containing ~120 μg of protein from 6 individual samples each one.

### Peptide fractionation by HPLC

Protein material was precipitated using methanol/chloroform. Pellets (~700 μg/pool) were dissolved in 100 mM Tris, pH 7.8, and 6 M urea. Reduction was performed by addition of DTT to a final concentration of 10 mM and incubation at 25°C for 1 h. Subsequent alkylation by 30 mM iodoacetamide was performed for 1 h in the dark. An additional reduction step was performed by 30 mM DTT, allowing the reaction to stand at 25°C for 1 h. Proteins were digested for 4 h with Lys-C (Promega) at 37°C (enzyme:protein, 1:140 w/w). The mixtures were then diluted to 0.6 M urea using MilliQ-water, and after trypsin addition (Promega) (enzyme:protein, 1:50 w/w), the sample was incubated at 37°C for 18 h. Digestion was quenched by acidification with acetic acid. The digestion mixture (~350 μg protein) was dried in a SpeedVac, reconstituted with 40ul of 5 mM ammonium bicarbonate (ABC) pH 9.8, and injected to an Ettan LC system with a high pH stable X-Terra RP18 column (C18; 2.1 mm × 150 mm; 3.5 μm) (Waters) at a flow rate of 40 μl/min. Peptides were eluted with a mobile phase B of 5–65% linear gradient over 35 min (A, 5 mM ABC in water at pH 9.8; B, 5 mM ABC in acetonitrile at pH 9.8). 16–18 fractions were collected, evaporated under vacuum and reconstituted into 15 μl of 2% acetonitrile, 0.1% formic acid, 98% water.

### Protein fractionation by isoelectric focusing (IEF)

Approximately 500 μg of each pooled sample was precipitated with methanol/chloroform. The pellet were re-suspended in 300 μl of IEF rehydration buffer (7 M urea, 2 M thiourea, 2% CHAPS, 50 mM DTT, 0.5% Bio-Lyte 3/10 ampholyte) and loaded on 17 cm pH 3–10 IPG strip in a focusing tray. Isoelectric focusing was performed on a Bio-Rad PROTEAN IEF system (Bio-Rad). Conditions for performing IEF were as follows: after rehydration of the IPG strip for 12 h at 50 V and 20°C, the run was started at 250 V for 15 min. followed by rapid voltage ramping to 8000 V without exceeding 50 A/strip. The IEF run was finished when the voltage reached 35,000 Vhr. The voltage was held at 500 V until the run was stopped. The IEF run was performed at 20°C. Each entire IPG strip was divided into 25 equal parts (~0.7 cm each) for in-gel reduction, alkylation, and digestion. In-gel tryptic digestion was performed with 20 ng/μl trypsin in 50 mM ammonium bicarbonate for 12 h at 37°C. The resulting tryptic peptides were extracted with 5% formic acid, 50% acetonitrile and 5% formic acid, 85% acetonitrile. Peptide mixtures were completely dried in a SpeedVac and resuspended in 20 μl 98 % MilliQ-H_2_O, 2 % acetonitrile, and 0.1 % formic acid.

### nanoLC-MS/MS analysis

For each fraction, a total volume of 5 μl of tryptic peptides was injected with a flow rate of 300 nL/min in a nanoLC Ultra1D plus (Eksigent). The column and the autosampler were maintained at a temperature of 40°C and 4°C respectively. A trap column Acclaim PepMap100 (100 μm × 2 cm; C18, 5 μm, 100 Å) and an analytical column Acclaim PepMap RSLC (75 μm × 15 cm, C18, 2 μm, 100 Å) (Dionex) were used following the next gradient: 0–1 min (2% Buffer B), 1–110 min (2–30% Buffer B), 110–120 min (30–40% Buffer B), 120–125 min (40-90% Buffer B), 125–130 min (90% Buffer B), 130–132 min (90-2% Buffer B) and 132–150 min (2% Buffer B) (Buffer B (100% acetonitrile, 0.1% formic acid), Buffer A (0.1% formic acid)). MS analysis was performed on a Q-TRAP 5500 system (ABSciex) with a NanoSpray® III ion source (ABSciex). The mass spectrometer was operated in positive ion mode at unit resolution. Each fraction was analyzed twice in technical replicates. For MS/MS analysis, survey scans were acquired from m/z 400 to 1000 with up to 6 precursors selected for MS/MS from m/z 230 to 1000 using dynamic exclusion, and the rolling collision energy was used to promote fragmentation. MS/MS data acquisition was performed using Analyst 1.5.2 (AB Sciex) and spectra files were processed through Protein Pilot™ Software (v.4.0.8085-ABSciex) using Paragon™ Algorithm (v.4.0.0.0) for database search against the concatenated target-decoy UniProt human database (Database: uniprot_sprot_20100622). For each substructure, 82–86 runs were processed sequentially with output files for each individual fraction and a merged, non-redundant output file generated for protein identifications. To minimize the false positive identification of proteins, an unused ProtScore ≥2 (corresponding to 99% confidence) was used as the qualification criteria. False discovery rate (FDR) was performed using a non-lineal fitting method [67] and displayed results were those reporting a protein level-FDR lower than 1%. The mass spectrometry proteomics data have been deposited to the ProteomeXchange Consortium (http://proteomecentral.proteomexchange.org) via the PRIDE partner repository [68] with the data set identifier PXD000742 for the CN, PXD000749 for the putamen, PXD000755 for the GPe, and PXD000757 for the GPi.

### Data handling and bioinformatic analysis

The proteins identified in this study were classified by DAVID (Database for Annotation, Visualization and Integrated Discovery) Bioinformatics Resources (v6.7) [46], where proteins are assigned in gene ontology (GO) terms, which rely on a controlled vocabulary for describing a protein in terms of its molecular function, biological process, or subcellular localization [69]. For functional annotation clustering, we set the following parameters: “Biological process and molecular function”, high stringency and EASE p-value <0.01. PANTHER (Protein annotation through evolutionary relationship) classification system (http://www.pantherdb.org/) [48] was used to perform proteome mapping analysis of BG proteins across specific-neuronal processes. Network analysis was performed submitting the corresponding protein IDs to the STRING (Search Tool for the Retrieval of Interacting Genes) software (v.9.1) (http://stringdb.org/) [34]. This is a large database of known and predicted protein interactions. Proteins were represented with nodes and the interactions with continuous lines to represent direct interactions (physical), while indirect ones (functional) were presented by interrupted lines. All the edges were supported by at least a reference from the literature or from canonical information stored in the STRING database. To minimize false positives as well as false negatives, only interactions tagged as “high confidence” (>0.7) in STRING database were considered. Protein/gene ID conversions were obtained using g:profiler (http://biit.cs.ut.ee/gprofiler/gconvert.cgi) [70] and Protein Identifier Cross-Reference tool (PICR) (http://www.ebi.ac.uk/Tools/picr/) [71].

## Additional files

Additional file 1:
**Proteins identified in Caudate Nucleus (CN).**
Additional file 2:
**Proteins identified in Putamen.**
Additional file 3:
**Proteins identified in Lateral Globus Pallidus (GPe).**
Additional file 4:
**Proteins identified in Medial Globus Pallidus (GPi).**
Additional file 5:
**Genetic Association Database results.**
Additional file 6:
**Functional analysis of basal ganglia proteins previously detected in CSF.**
Additional file 7: Figure S1. General characteristics of the subjects included in the proteomic study. **Figure S2.** A representative image of BG structures analyzed in this study. **Figure S3.** Basal ganglia protein interactome network. **Figure S4.** Representative biological process categories from each cluster in striatal and pallidal structures. **Figure S5.** Representative molecular function categories from each cluster in striatum and globus pallidus. **Figure S6A.** Cellular Component Ontology for the striatal and pallidal proteomic expression profiles. Classification of BG proteomes based on cellular localization. **Figure S6B.** Cellular Component Ontology for the striatal and pallidal proteomic expression profiles. Neuron-specific localization detected by DAVID software. Additional file 8:
**Protein-protein interactions for the common proteome detected in Gpe, Gpi, CN y putamen using the STRING software.**
Additional file 9:
**Compilation of synaptic proteome databases. Synaptic proteome detected in basal ganglia structures.**
Additional file 10:
**Significantly enriched categories in Biological Process Ontology.**
Additional file 11:
**Significantly enriched categories in Molecular Function and Cellular Component Ontologies.**
Additional file 12:
**KEGG pathways analysis of basal ganglia proteome datasets.**
Additional file 13:
**Striatal and pallidal proteome distributions across specific reactions using the PANTHER classification system.**
Additional file 14:
**Anatomical correlation between pallidal and striatal proteomic expression profiling and human brain transcriptome.**
Additional file 15:
**Immunohistochemical analysis of Excitatory Amino Acid Transporter 2 and A-Kinase anchor protein 12 in striatal and pallidal structures.**

## Abbreviations

BG: Basal ganglia
CN: Caudate nucleus
IEF: Isoelectric focusing
SN: substantia nigra
GP: Globus pallidus
GPi: Medial globus pallidus
GPe: Lateral globus pallidus
STN: Subthalamic nucleus
MS: Mass spectrometry
NanoLC-MS/MS: Nano-liquid chromatography tandem mass spectrometry
2-DE: Bidimensional electrophoresis
