# Supplementary material for: Anatomo-proteomic characterization of human basal ganglia: focus on striatum and globus pallidus
Source: Mol Brain. 2014 Nov 18;7:83. doi: 10.1186/s13041-014-0083-9 (PMC4236423; doi:10.1186/s13041-014-0083-9)
Supplement: Additional file 7: Figure S1. — General characteristics of the subjects included in the proteomic study. Figure S2. A representative image of BG structures analyzed in this study. Figure S3. Basal ganglia protein interactome network. Figure S4. Representative biological process categories from each cluster in striatal and pallidal structures. Figure S5. Representative molecular function categories from each cluster in striatum and globus pallidus. Figure S6A. Cellular Component Ontology for the striatal and pallidal proteomic expression profiles. Classification of BG proteomes based on cellular localization. Figure S6B. Cellular Component Ontology for the striatal and pallidal proteomic expression profiles. Neuron-specific localization detected by DAVID software. [file 13041_2014_83_MOESM7_ESM.pdf]

Anatomo-Proteomic Characterization of Human Basal Ganglia: Focus on striatum and Globus Pallidus

Joaquín Fernández-Irigoyen[1], María Victoria Zelaya[2], Teresa Tuñon[3], Enrique Santamaría[1*]

[1]Proteomics Unit, Clinical Neuroproteomics Group, Navarrabiomed, Fundación Miguel Servet, Pamplona, Spain
[2]Neurological Tissue Bank, Navarrabiomed, Fundación Miguel Servet, 31008 Pamplona, Spain
[3]Pathological Anatomy Department, Navarra Hospital Complex, Pamplona, Spain

Case	Sex	Age	Macroscopy analysis	PMI (hours)	Immunohistochemistry analysis (Tau, β-amyloide, TDP43, PrP, α-syn, Ub, αβ cryst)	Diagnostic
BCN 34	M	54	normal	3.25	negative	control
BCN 115	M	54	normal	> 8	negative	control
BCN 169	M	41	normal	3.5	negative	control
BCN 174	M	29	normal	7	negative	control
BCN 251	M	53	normal	7	negative	control
BCN 294	M	61	normal	8	negative	control

Supporting figure 1: General characteristics of the subjects included in the proteomic study

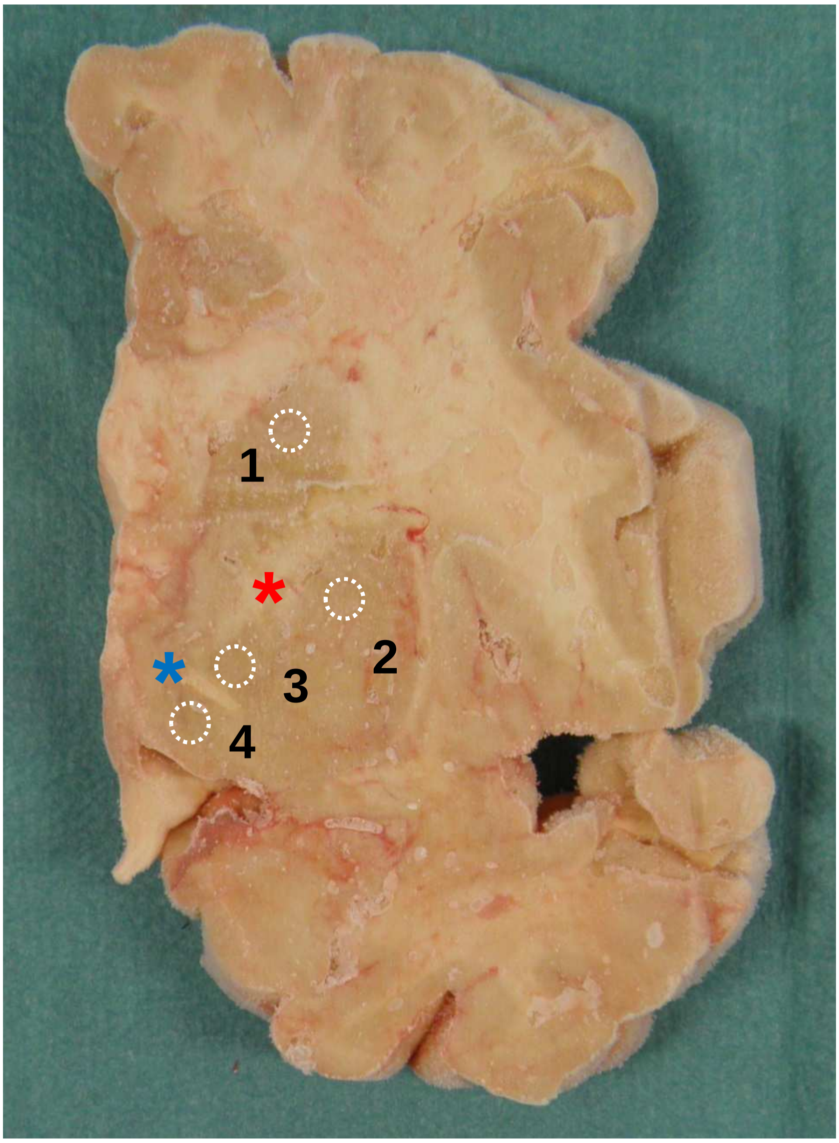

Supporting figure 2: A representative image of BG structures analyzed in this study. The internal capsule and the medial medullary lamina are indicated as a reference. 1) Caudate nucleus, 2) Putamen, 3) Lateral globus pallidus (external), 4) Medial globus pallidus (internal)

*) internal capsule
*) Medial medullary lamina

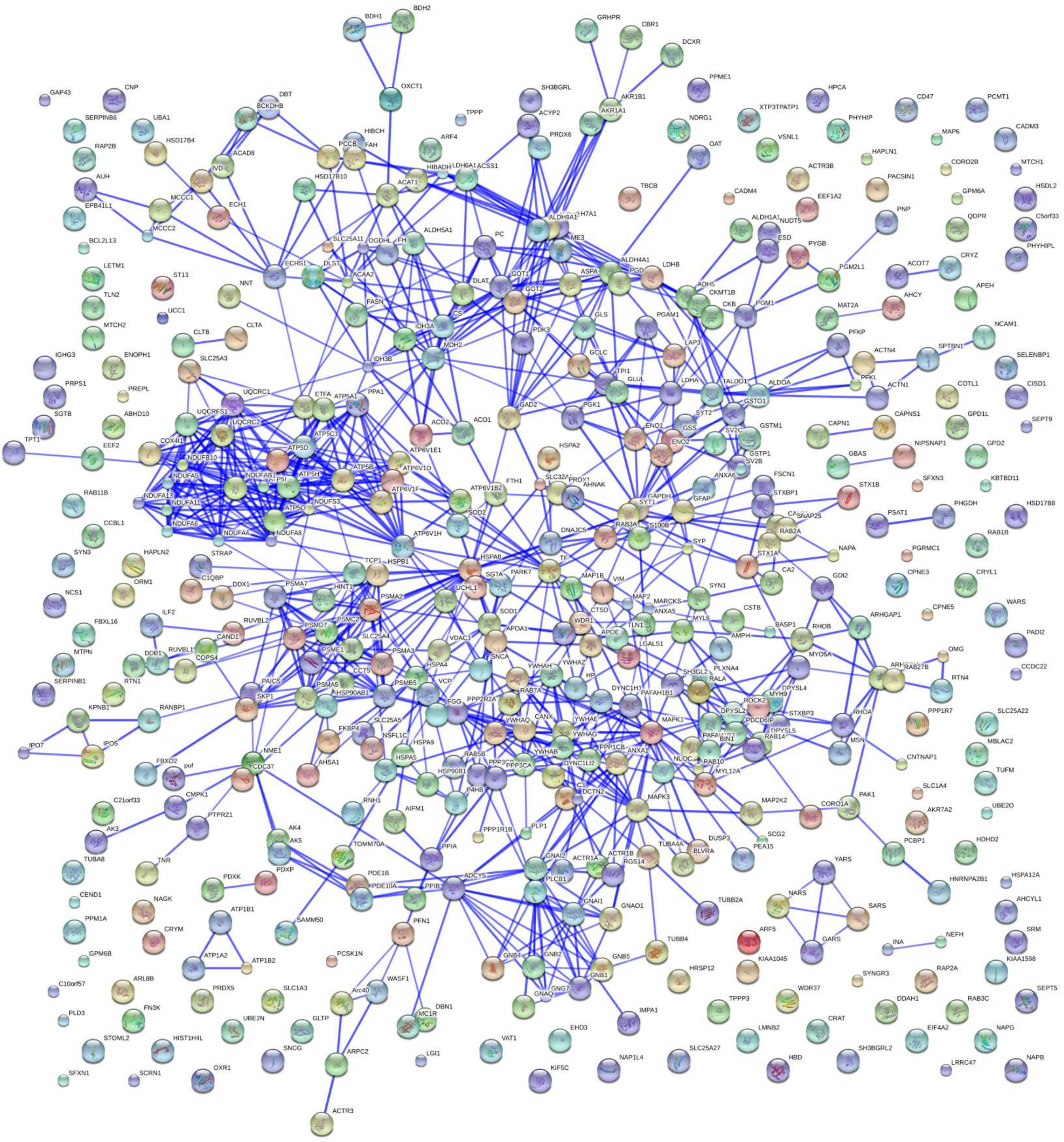

Supporting figure 3: BG interactome network.

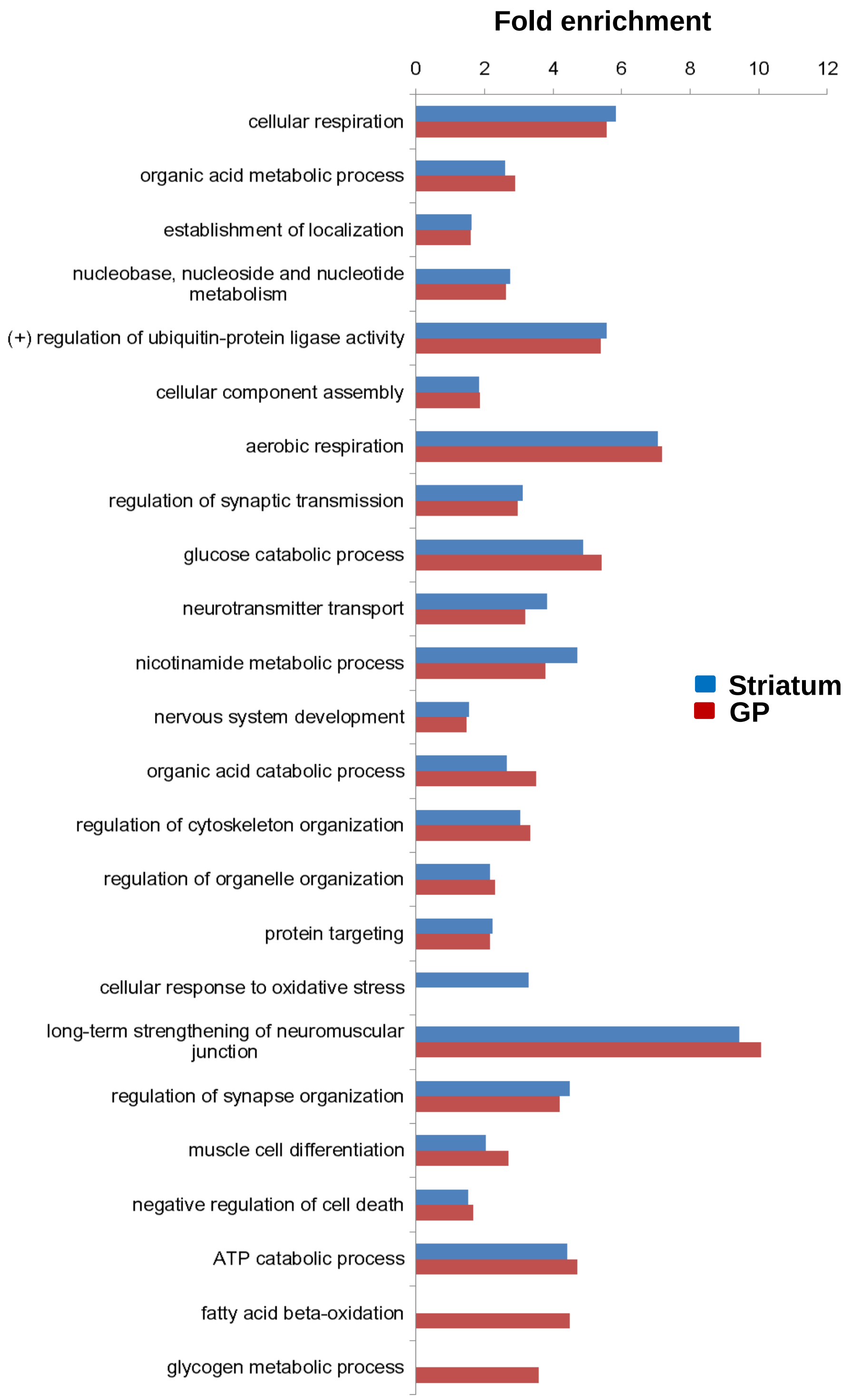

Supporting figure 4: Biological Process Ontology for the striatal and pallidal proteomic expression profiles. Representative enriched GO biological process terms from 24 significantly annotation clusters are shown (fold enrichment > 1.5, EASE p-value <0.01). Fold enrichment refers to the number of relevant basal ganglia protein species represented in each category relative to random expression of all genes in the human genome. A complete characterization of each cluster is shown in supplementary table 9.

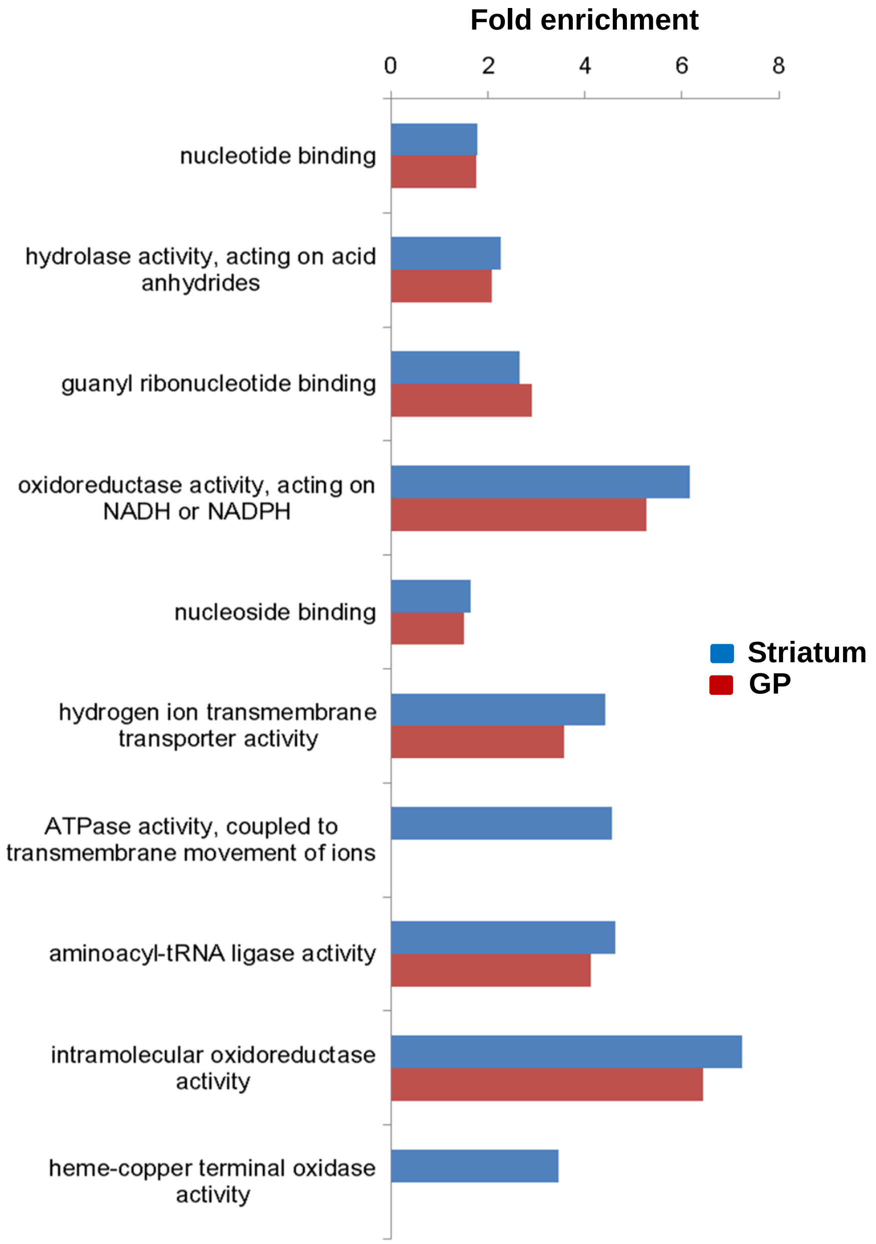

Supporting figure 5: Molecular Function Ontology for the striatal and pallidal proteomic expression profiles. Representative enriched GO molecular function terms from 10 significantly annotation clusters are shown (fold enrichment > 1.5, EASE p-value <0.01). Fold enrichment refers to the number of relevant basal ganglia protein species represented in each category relative to random expression of all genes in the human genome. A complete characterization of each cluster is shown in supplementary table 10.

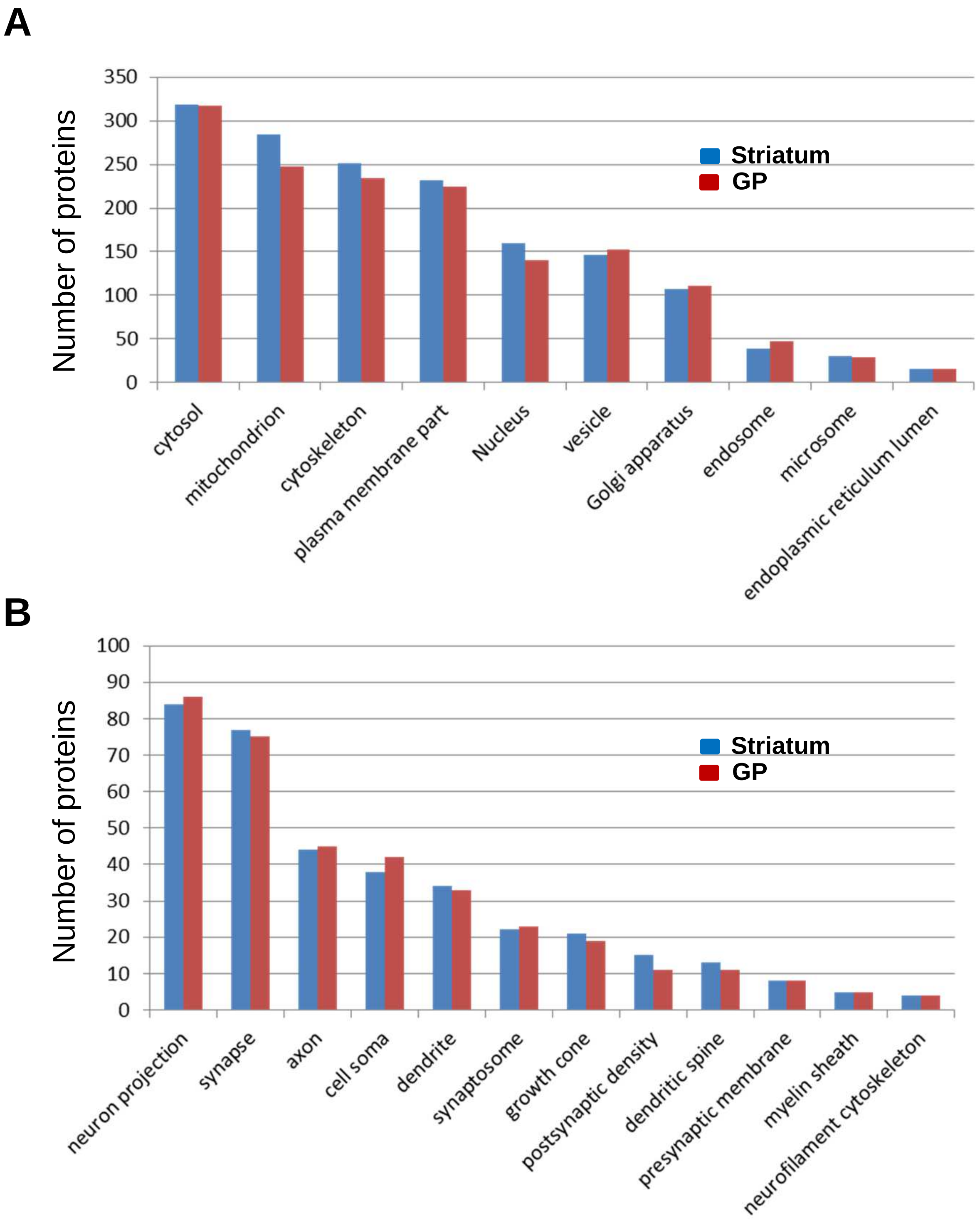

Supporting figure 6: Cellular Component Ontology for the striatal and pallidal proteomic expression profiles. A) Classification of BG proteomes based on cellular localization. B) Neuron-specific localization detected by DAVID software (see supplementary table 10).
