# Supplementary material for: Anatomo-proteomic characterization of human basal ganglia: focus on striatum and globus pallidus
Source: Mol Brain. 2014 Nov 18;7:83. doi: 10.1186/s13041-014-0083-9 (PMC4236423; doi:10.1186/s13041-014-0083-9)
Supplement: Additional file 15: — Immunohistochemical analysis of Excitatory Amino Acid Transporter 2 and A-Kinase anchor protein 12 in striatal and pallidal structures. [file 13041_2014_83_MOESM15_ESM.pdf]

Supplementary figure 7: Immunohistochemistry analysis of Excitatory amino acid transporter 2 and A-Kinase anchor protein 12 in human striatum and globus pallidus derived from three independent subjects. The excitatory amino acid transporter 2 and A-Kinase anchor protein 12 were exclusively detected in pallidal and striatal structures respectively using proteomics. However, immunohistochemical analysis reveals that both proteins are expressed in both striatum and globus pallidus.

Case 1: male, 61 years
Excitatory amino acid transporter 2
1H8 antibody; MA5-12360 (Pierce)
Dilution: 1:10

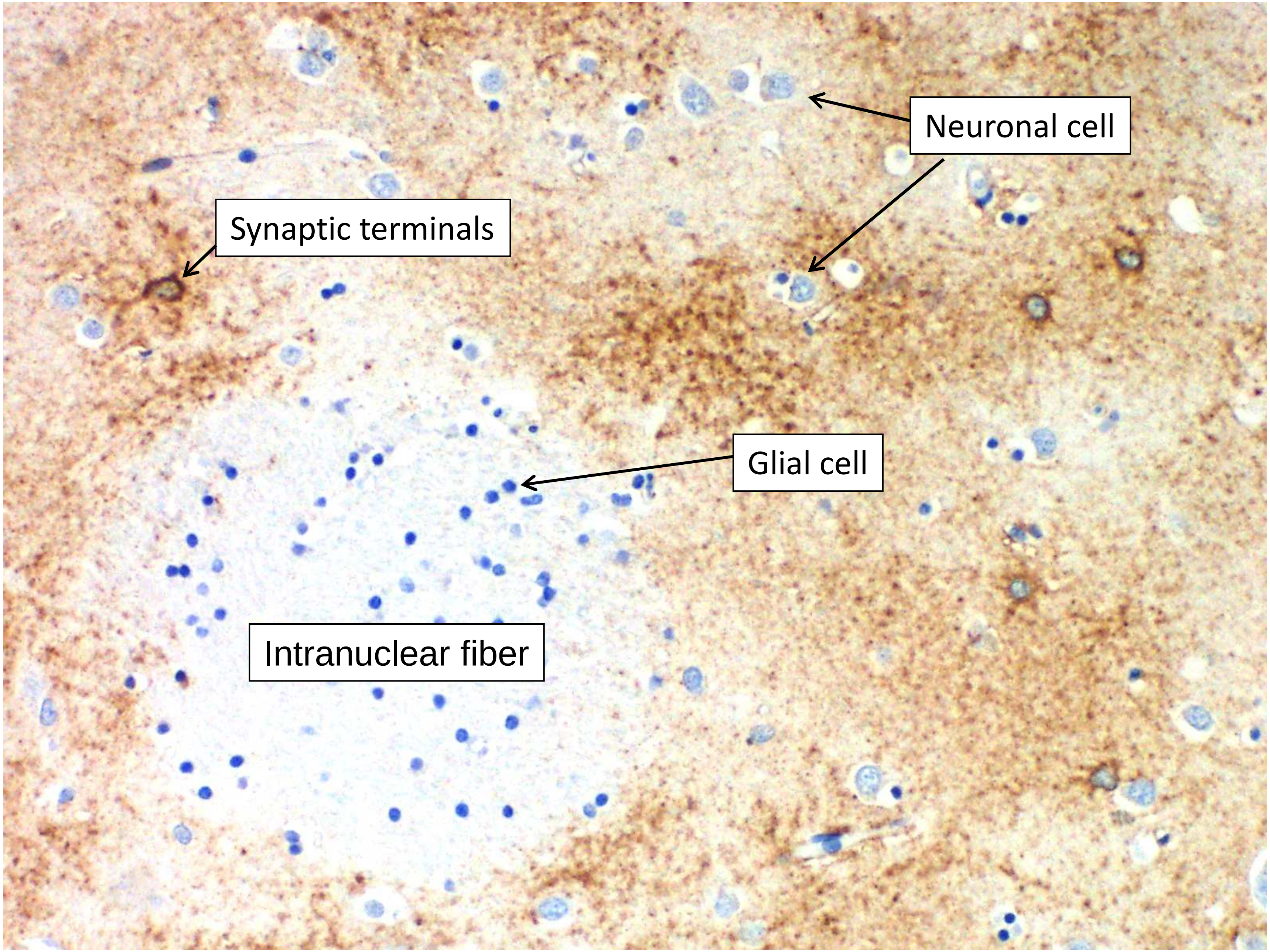

Caudate Nucleus: Diffuse immunostaining of 1H8 antibody (20X). Synaptic staining pattern of neuropil and synaptic terminals. Negative staining of intranuclear fiber, neuronal an glial cells.

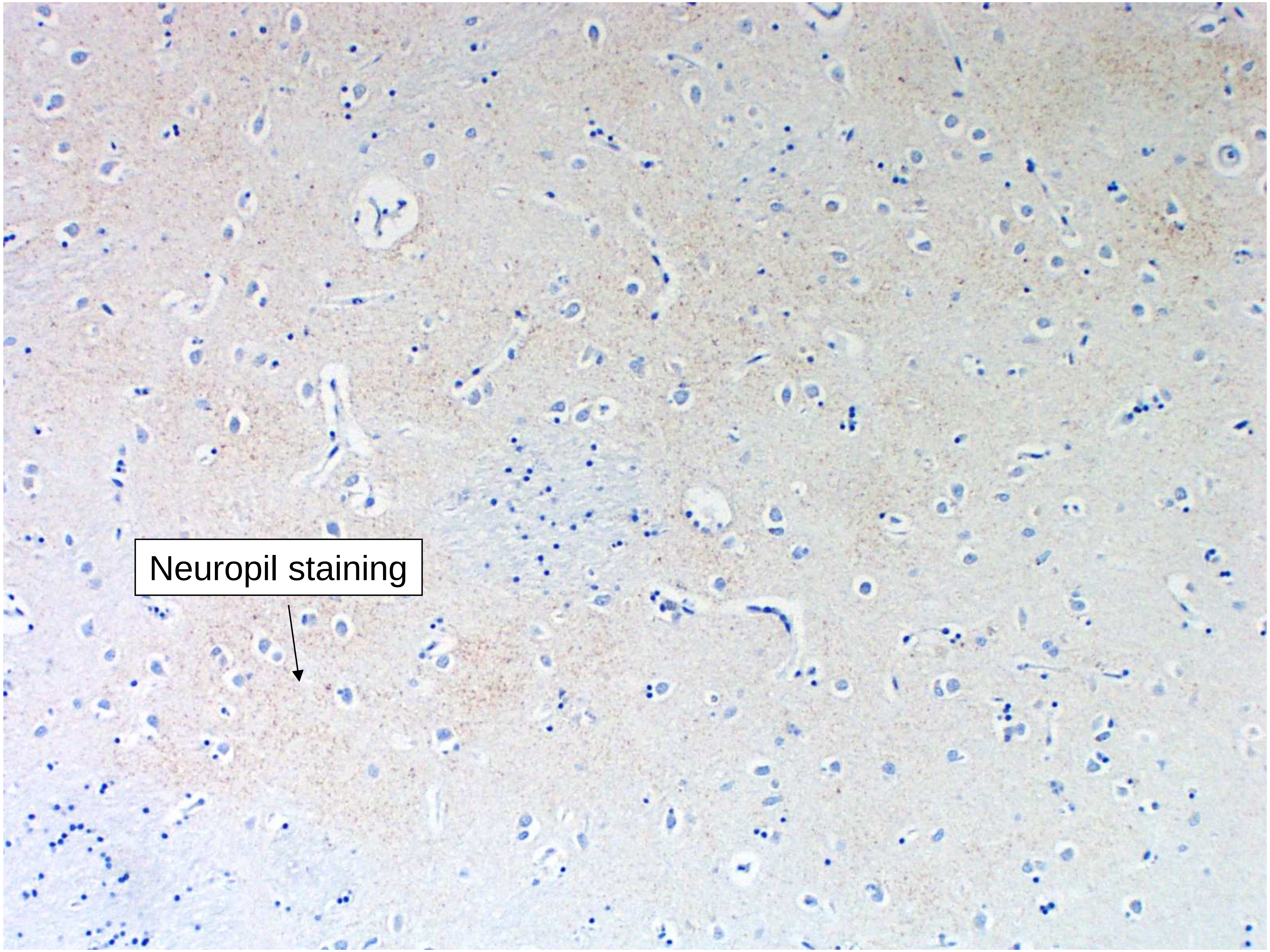

Putamen: Lower intensity of 1H8 staining than in caudate nucleus. Negative staining of neuron and glial cells (20x).

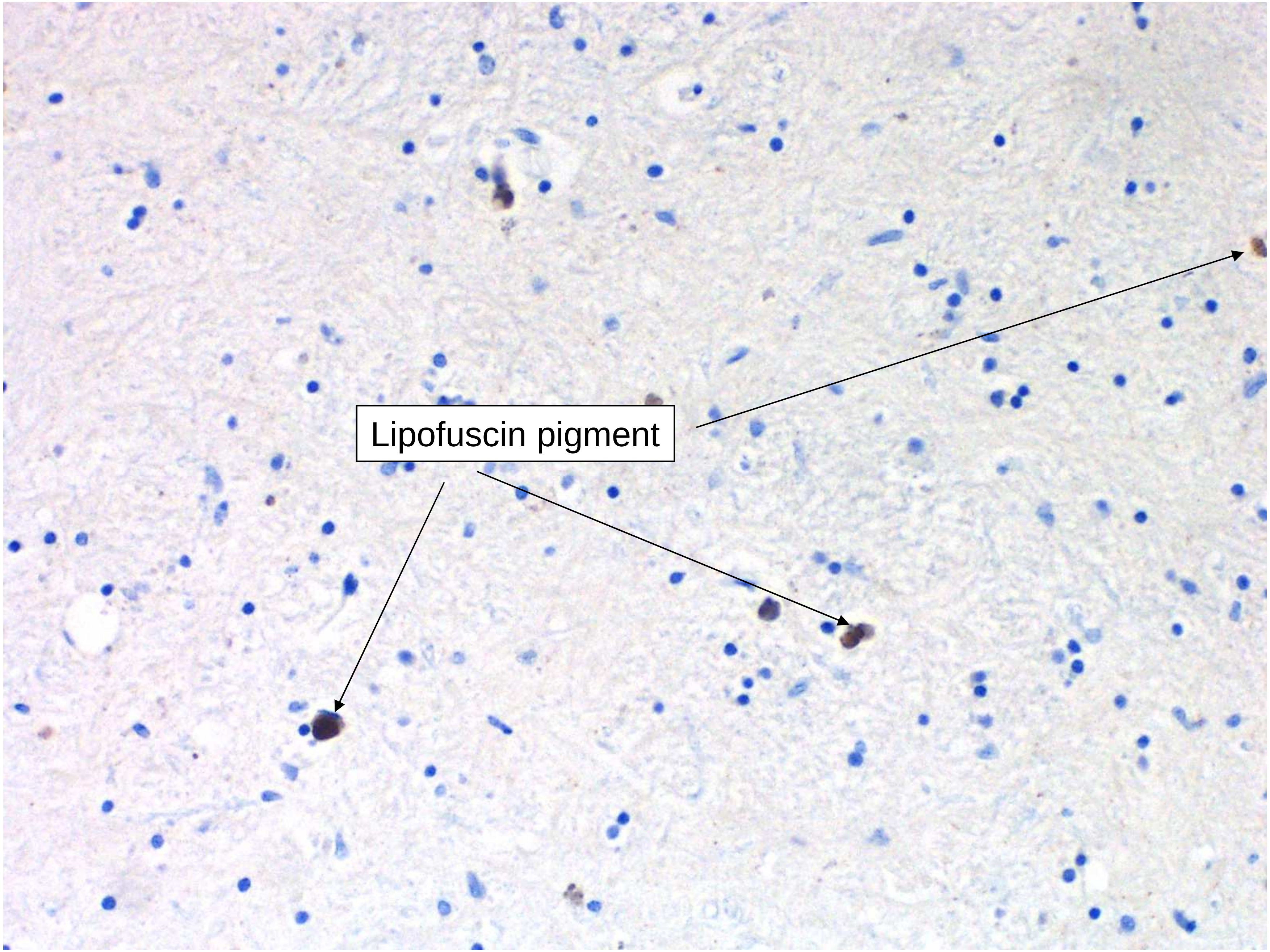

Globus Pallidus: Negative inmunostaining of 1H8 (40x). Presence of lipofuscin pigments

Case 2: male, 54 years
Excitatory amino acid transporter 2
1H8 antibody; MA5-12360 (Pierce)
Dilution: 1:10

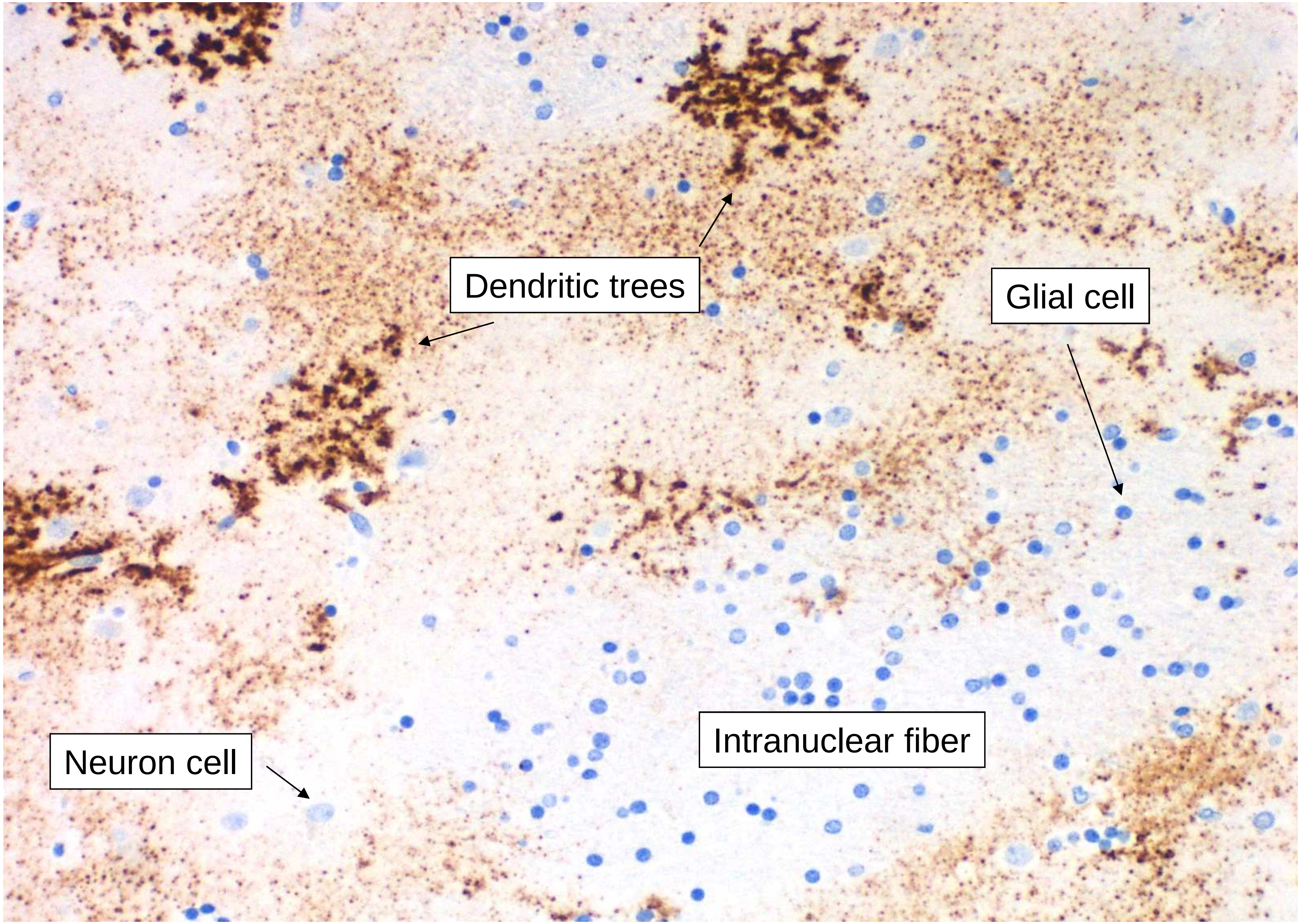

Caudate nucleus: Granular staining pattern of neuropil with 1H8 (20x). Positive stainning at dendritic trees and negative in neurons and glial cells.

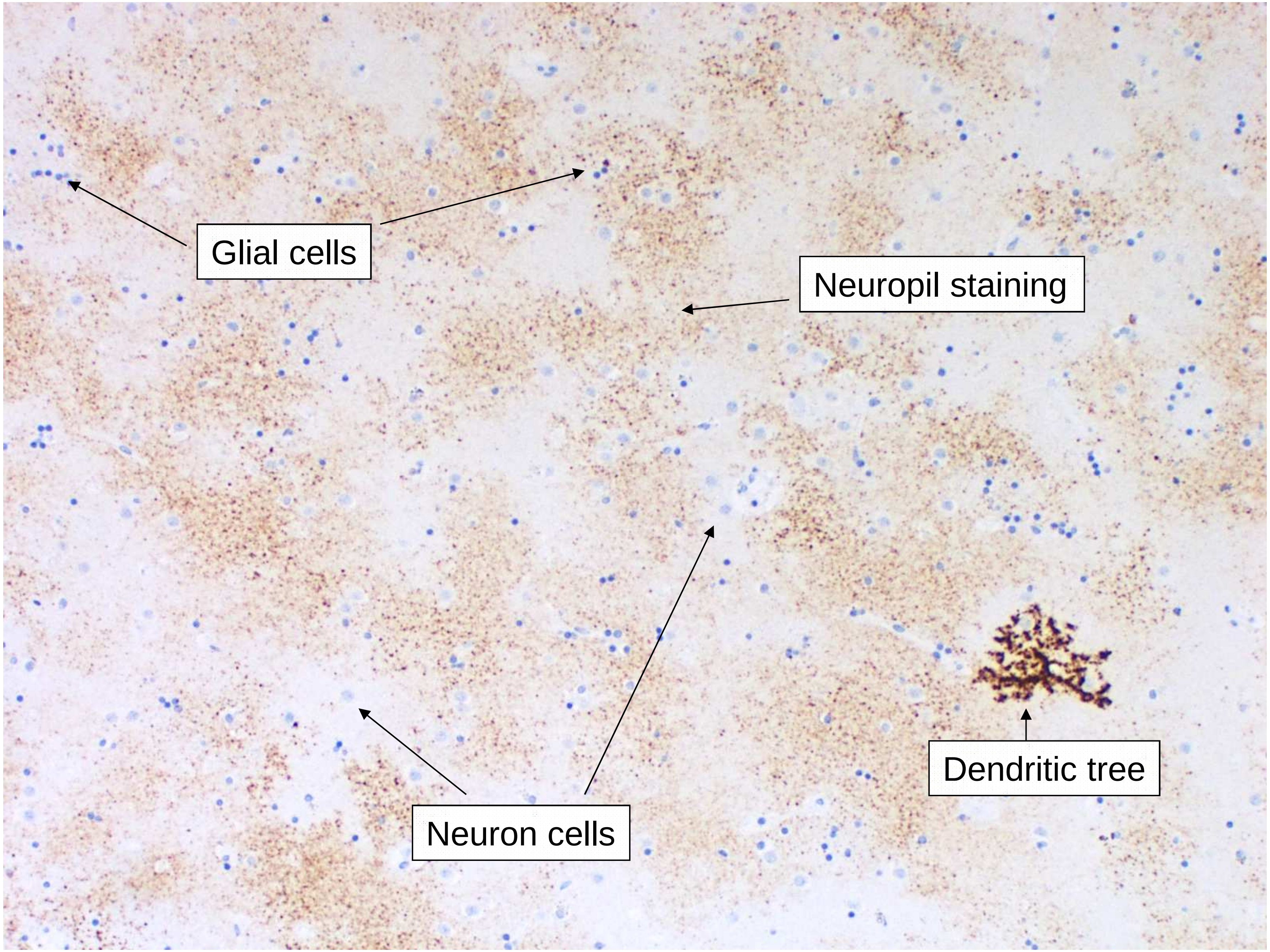

Putamen: Lower inmunostained deposit of 1H8 antibody than in caudate nucleus. Occasional dendritic trees and diffuse granular deposit of neuropil. Neuronal and glial cells are negative (10X)

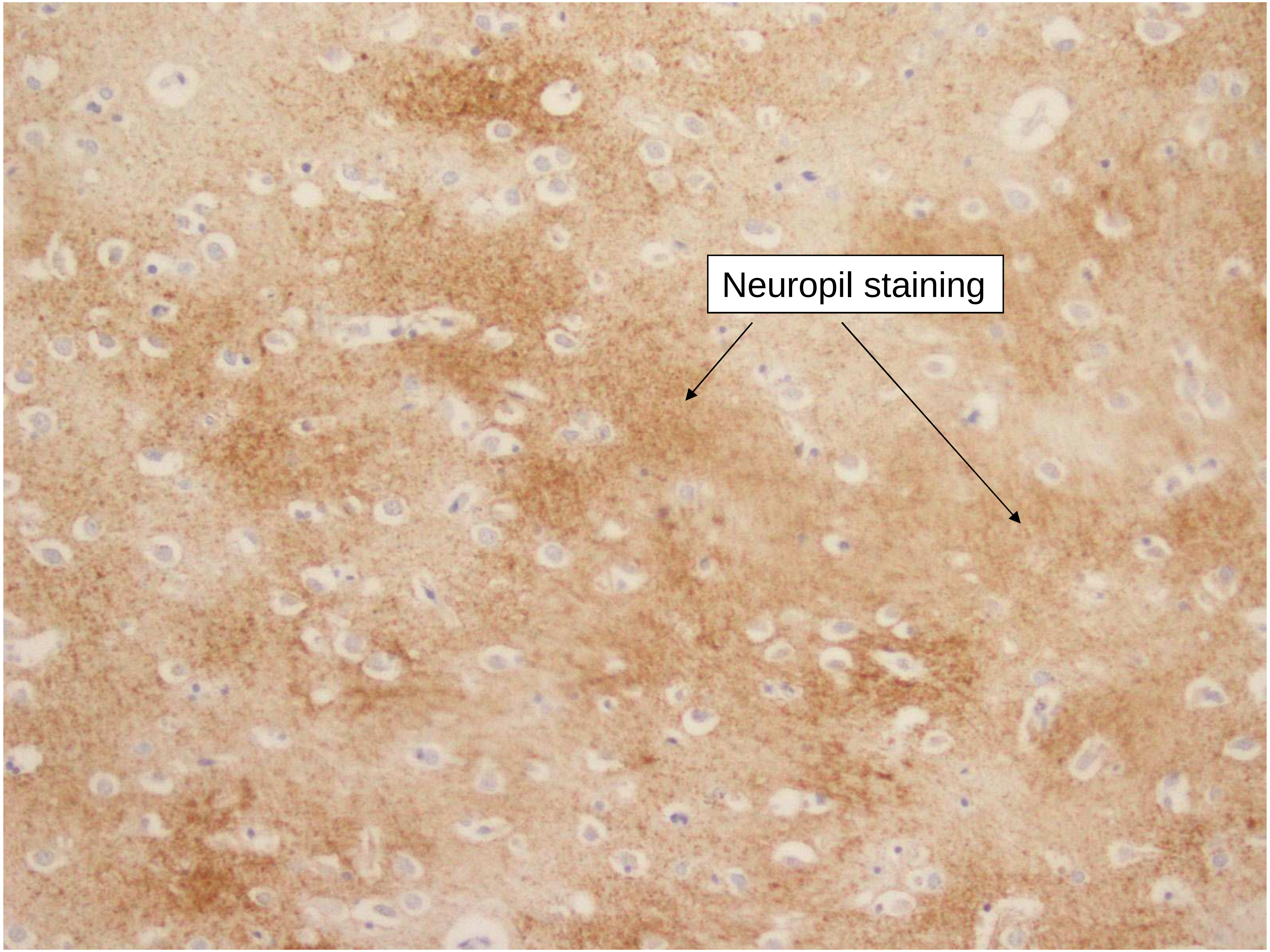

Globus Pallidus: Positive inmunostaining of 1H8 of neuropil. Negative staining of neurons and glial cells (20x).

Case 3: male, 41 years
Excitatory amino acid transporter 2
1H8 antibody; MA5-12360 (Pierce)
Dilution: 1:10

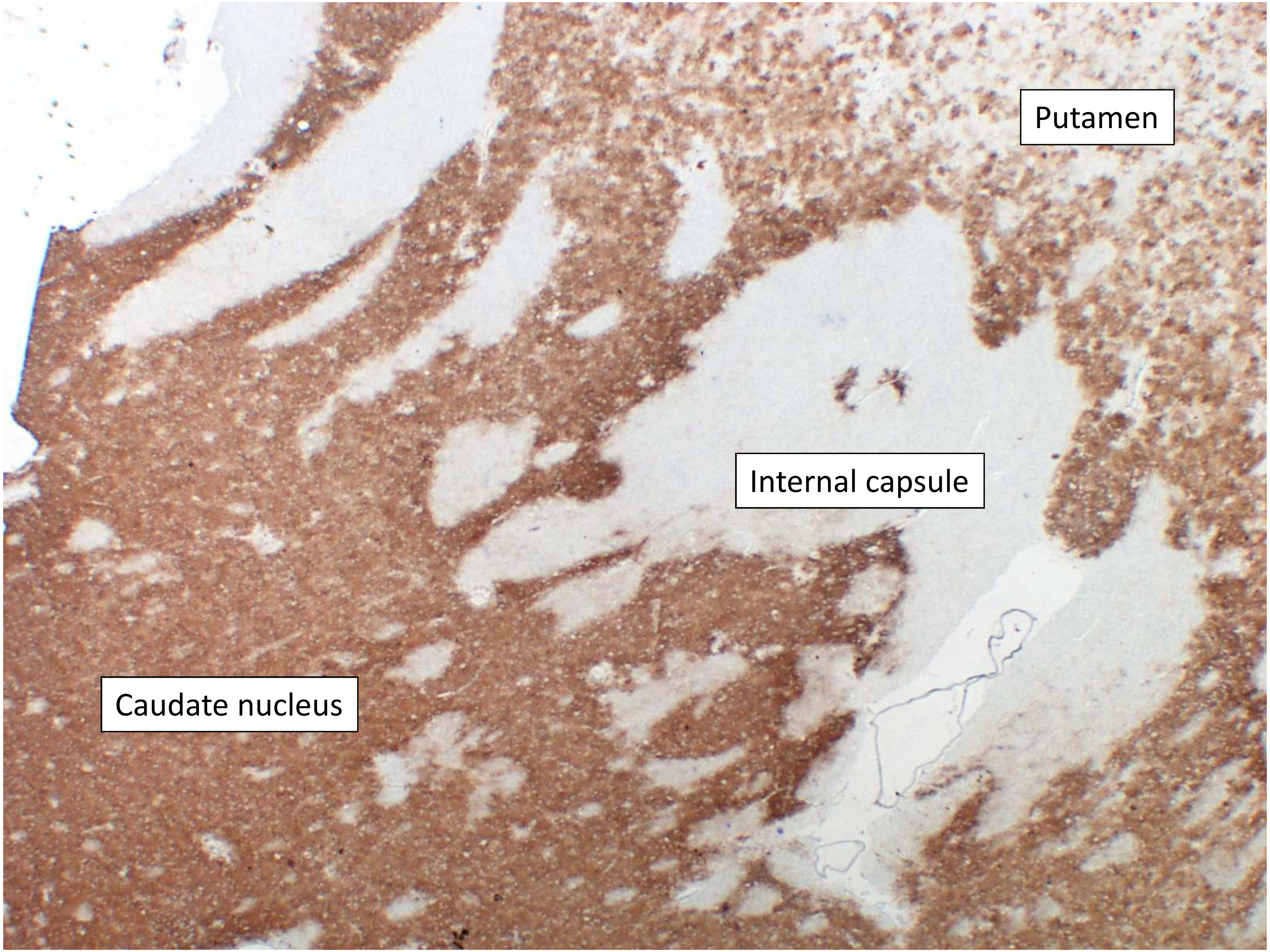

Immunohistochemical expression of 1H8 in basal ganglia (4x)

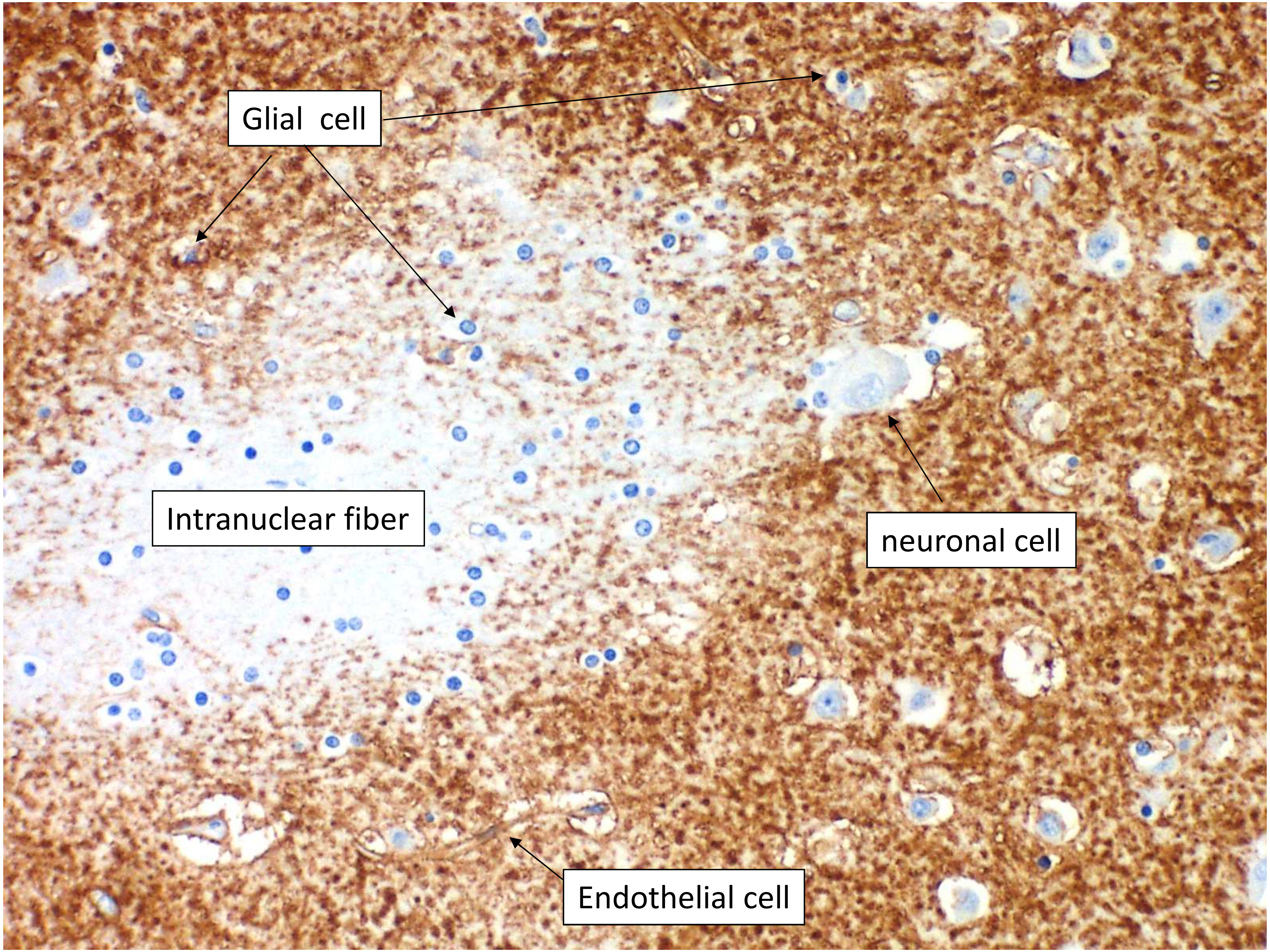

Caudate nucleus: Difuse immunostaining of 1H8 in neuropil, endothelial cells and partial in cytoplasm of neuron and glial cells (20X).

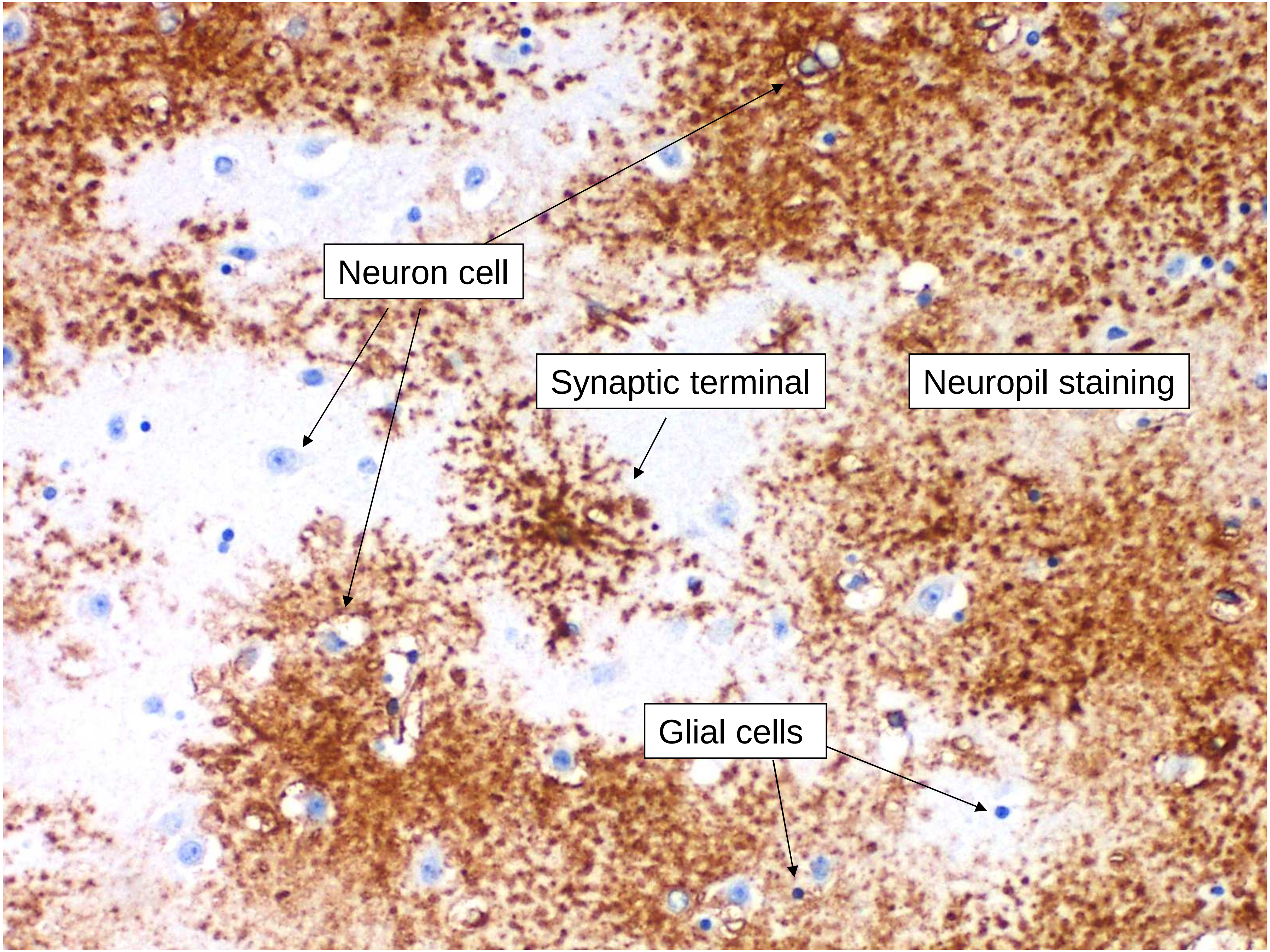

Putamen: Partial staining of different areas (20X). Some structures are negative and some positive.

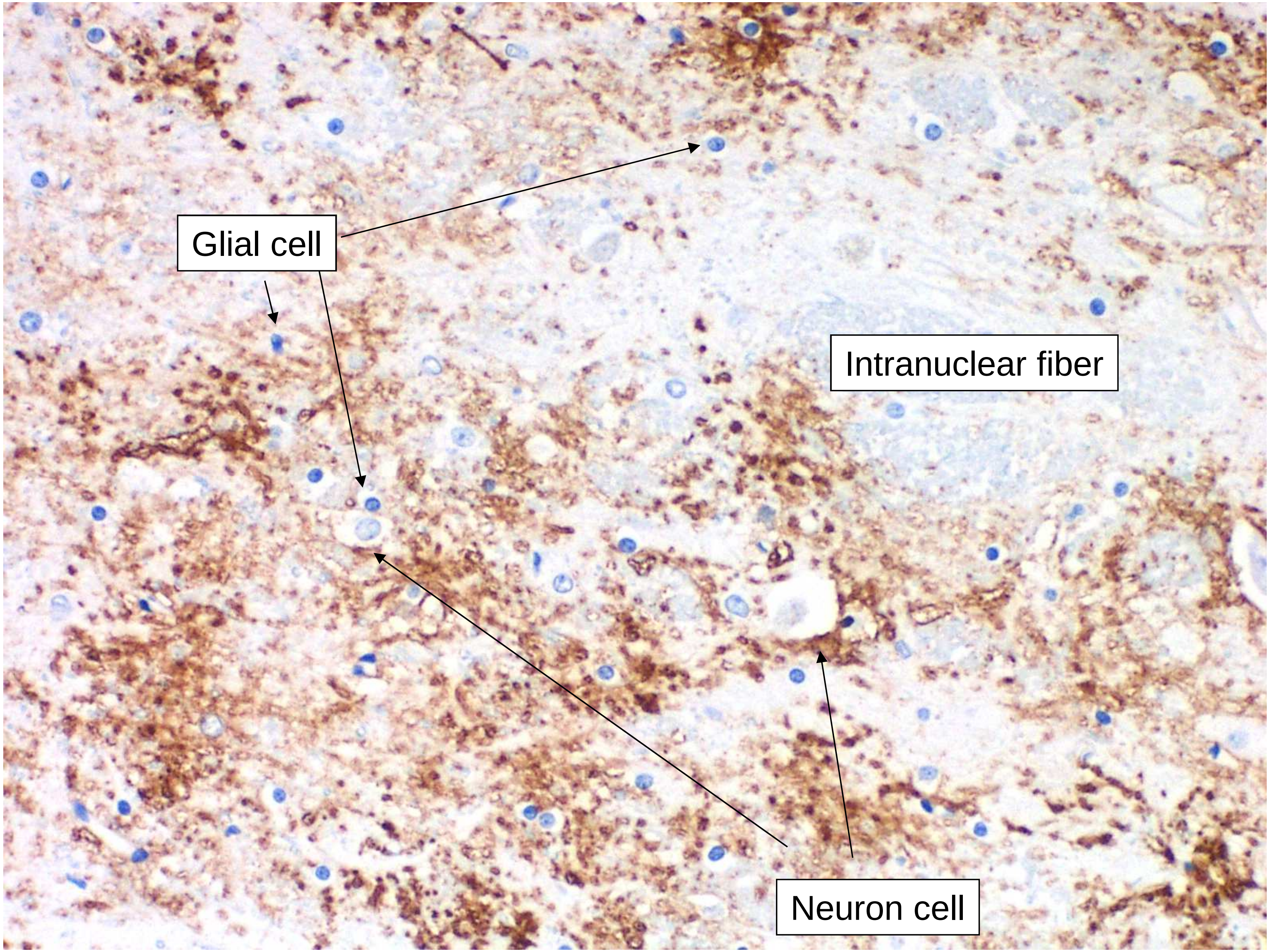

Globus pallidus: Intense inmunestained deposit of 1H8 antibody. Synaptic staining pattern. Negative glial cells and myelin fibers (20x).

Case 1: male, 61 years
A-kinase anchor protein 12
AKAP12 antibody; PA5-21759 (Pierce)
Dilution: 1:500

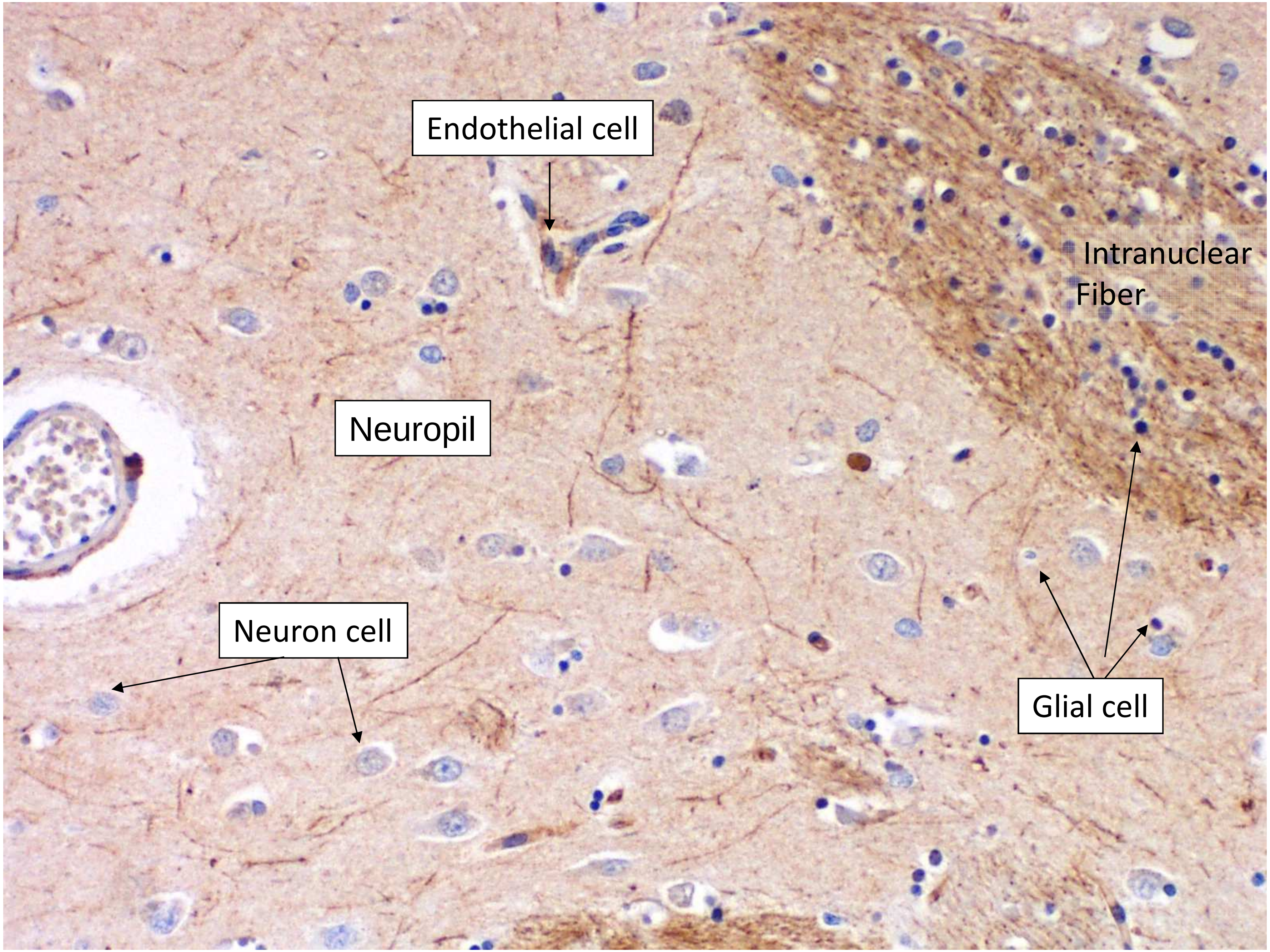

Caudate nucleus: Diffuse inmunostained of AKAP 12 antibody. Positive staining of neuropil, endothelial cells and intranuclear fibers . Partial staining of neuronal and glial cells (20X).

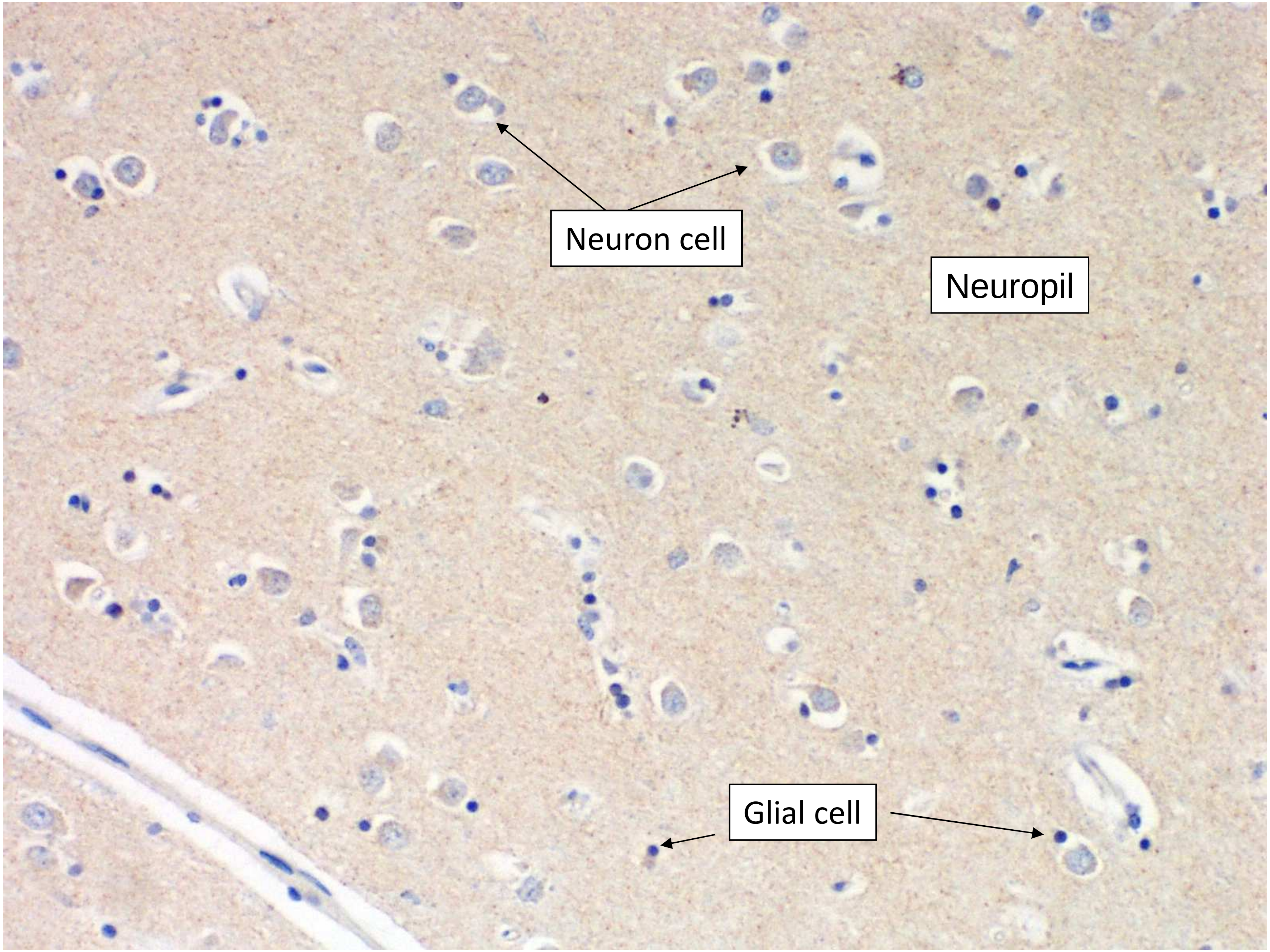

Putamen: Lower staining than caudate nucleus of AKAP 12 (40X)

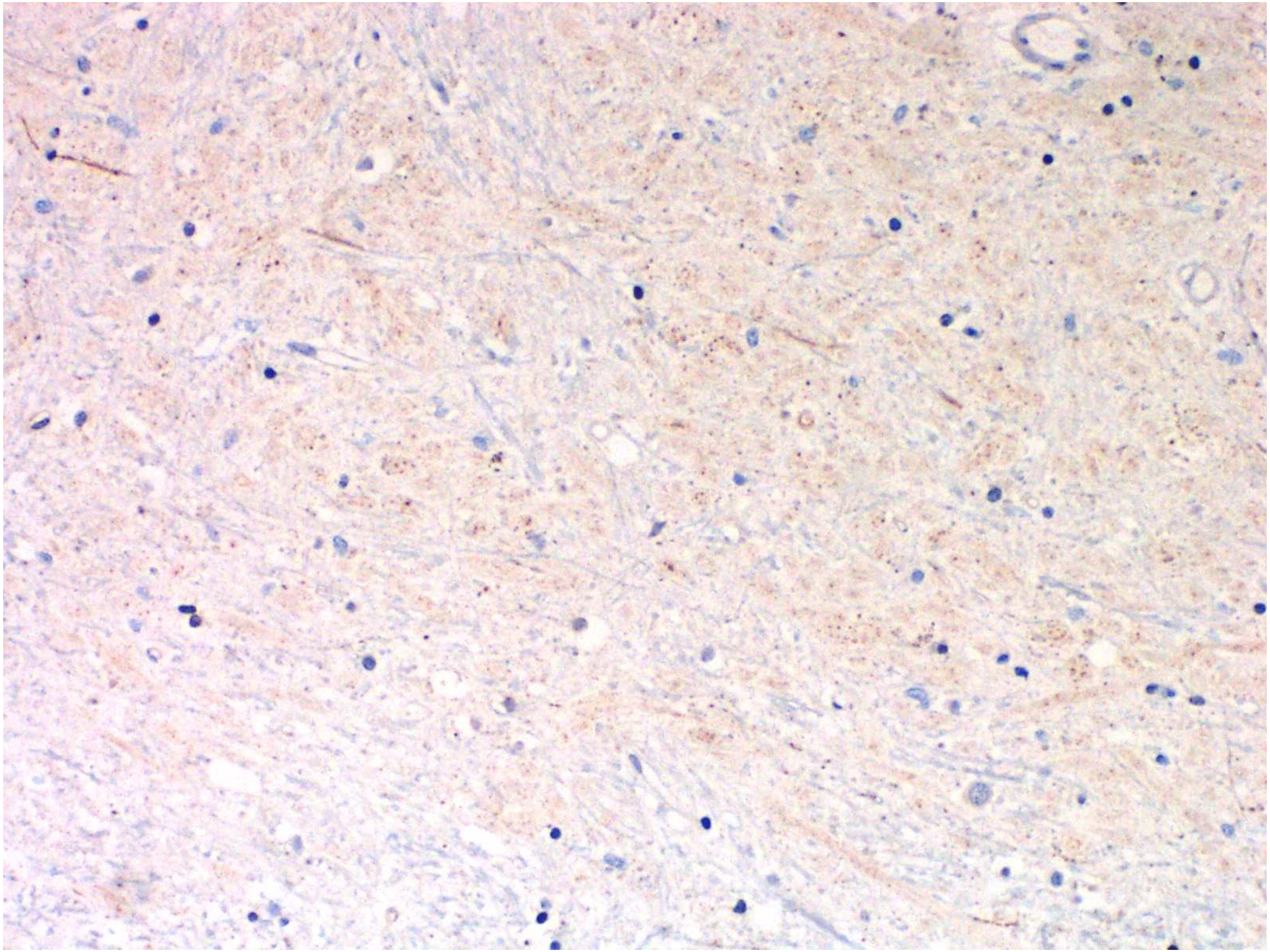

Globus pallidus: Granular staining pattern of AKAP 12 (20X).

Case 2: male, 54 years
A-kinase anchor protein 12
AKAP12 antibody; PA5-21759 (Pierce)
Dilution: 1:500

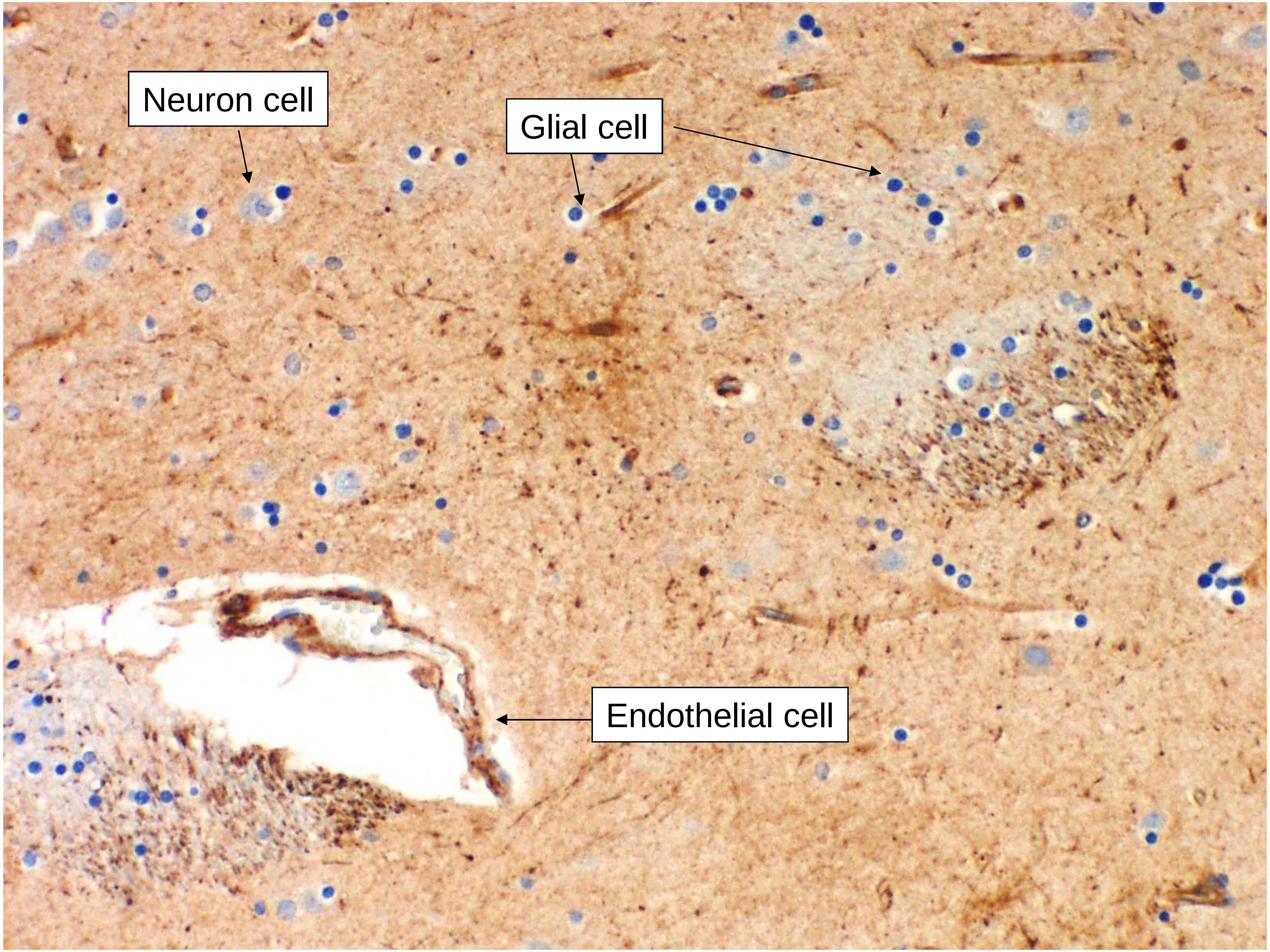

Caudate nucleus: Diffuse immunostaining of AKAP 12. Positive staining in neuropil, endothelial cells, cytoplasm of neuronal and glial cells (20X).

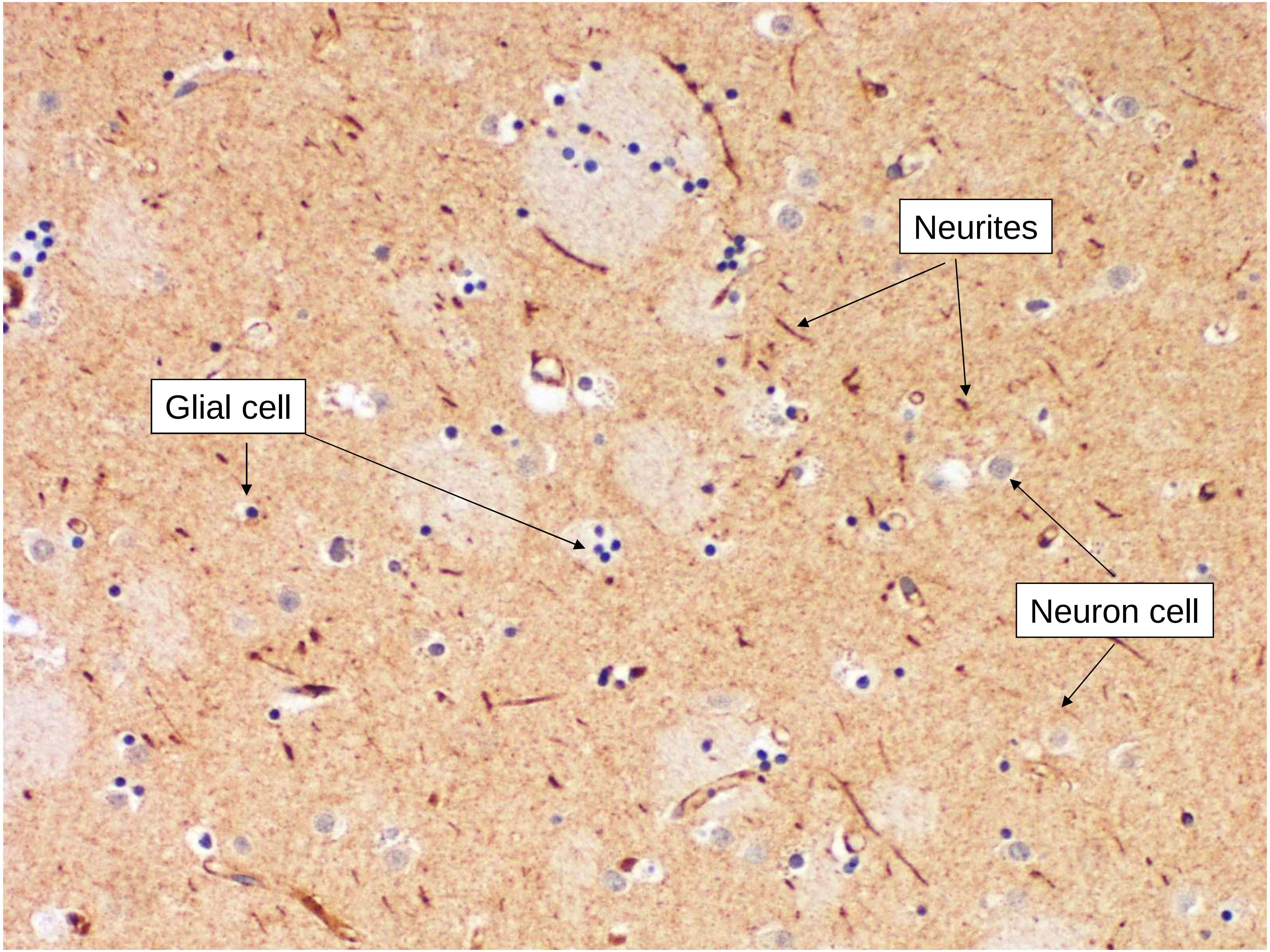

Putamen: Diffuse immunostaining similar than caudate nucleus. (20X)

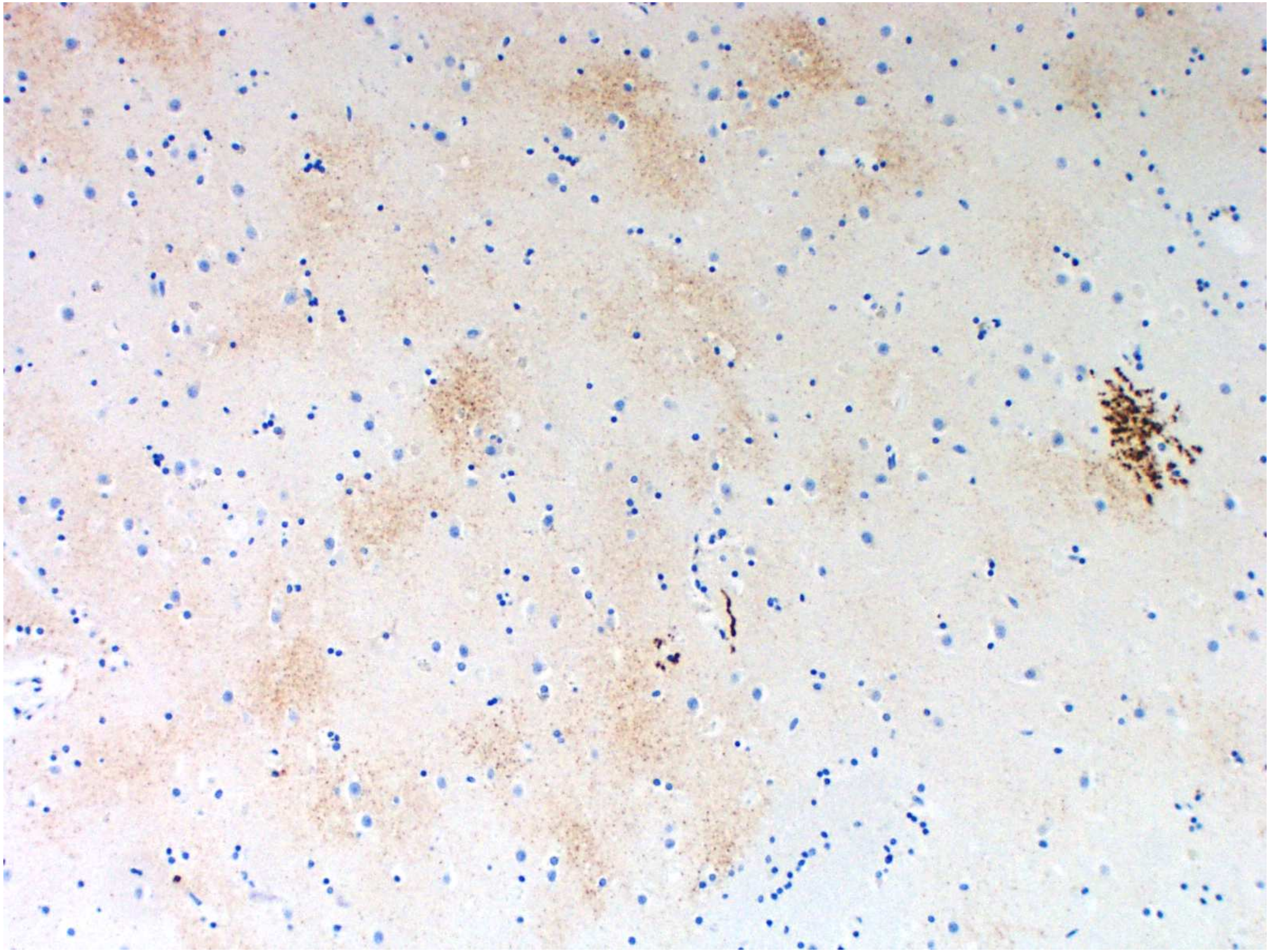

Globus Pallidus: Partial deposit of AKAP 12. Positive immunostaining in neuropil, occasional dendritic tree and neurites . Negative deposit in glial and neuronal cells and fibers (10X).

Case 3: male, 41 years
A-kinase anchor protein 12
AKAP12 antibody; PA5-21759 (Pierce)
Dilution: 1:500

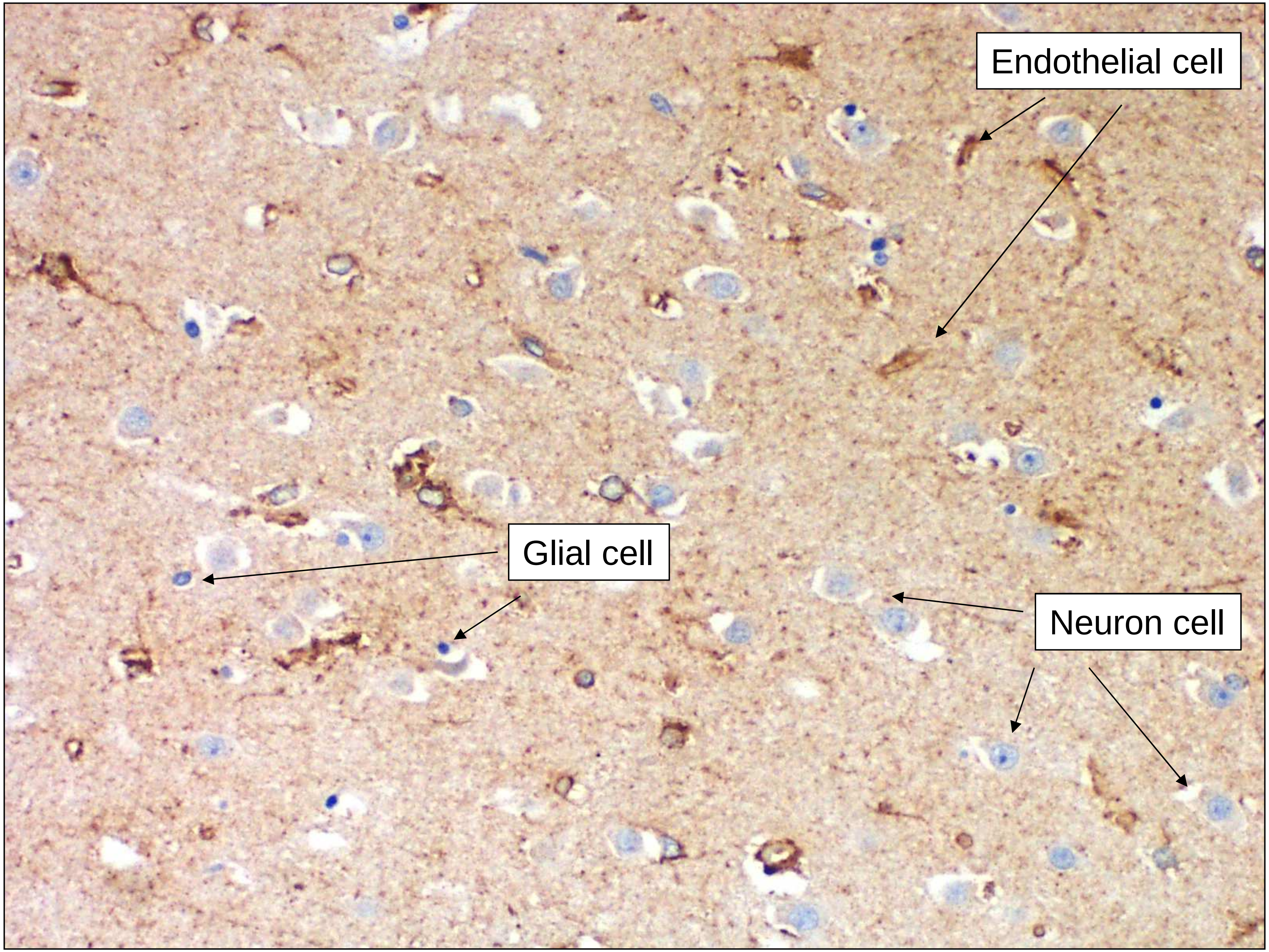

Caudate nucleus: Diffuse immunostaining of AKAP12 in neuropil, cytoplasm of endothelial, neuronal, and glial cells. (20X)

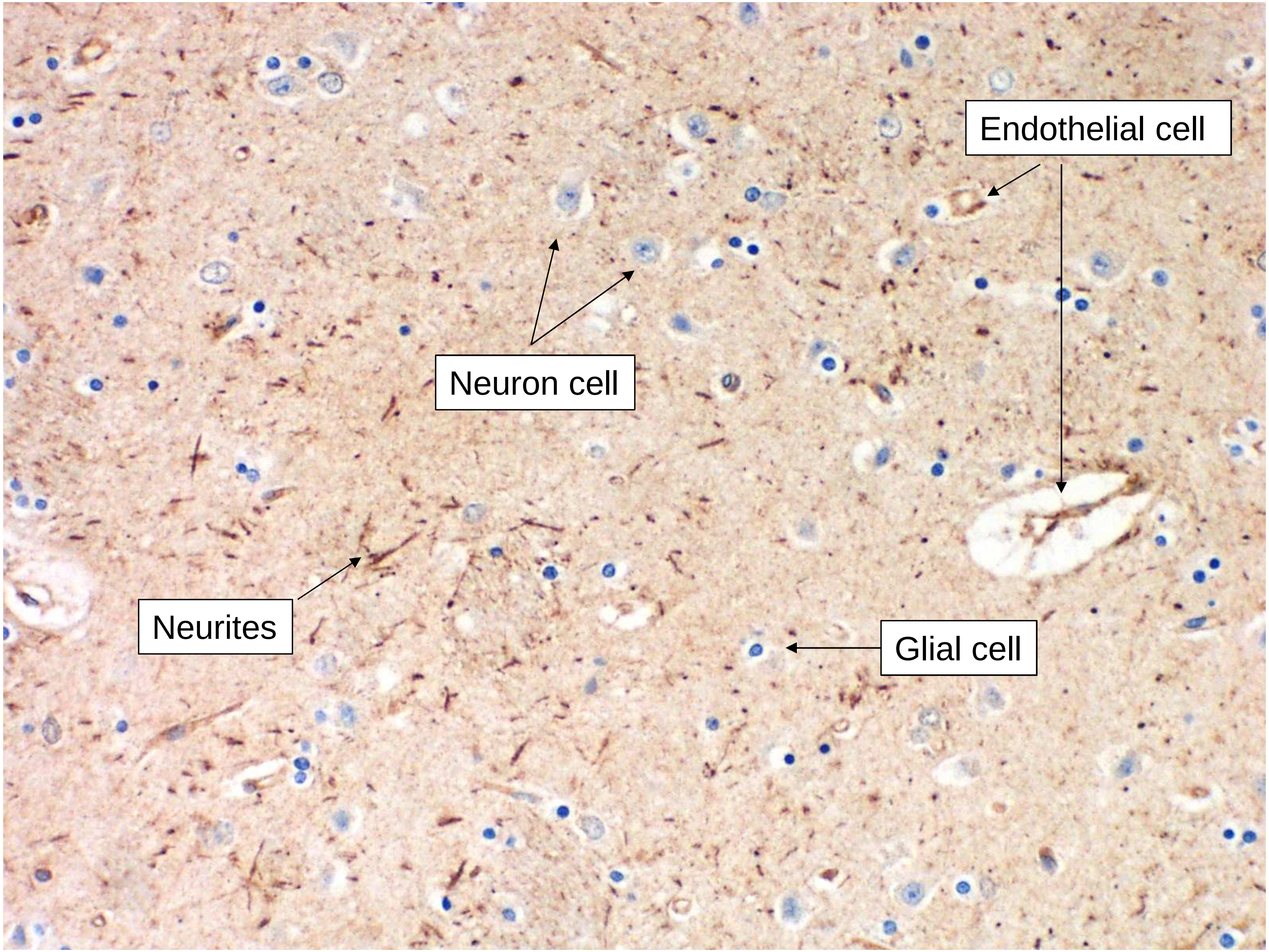

Putamen: Similar staining as caudate nucleus but lower intensity (20X)

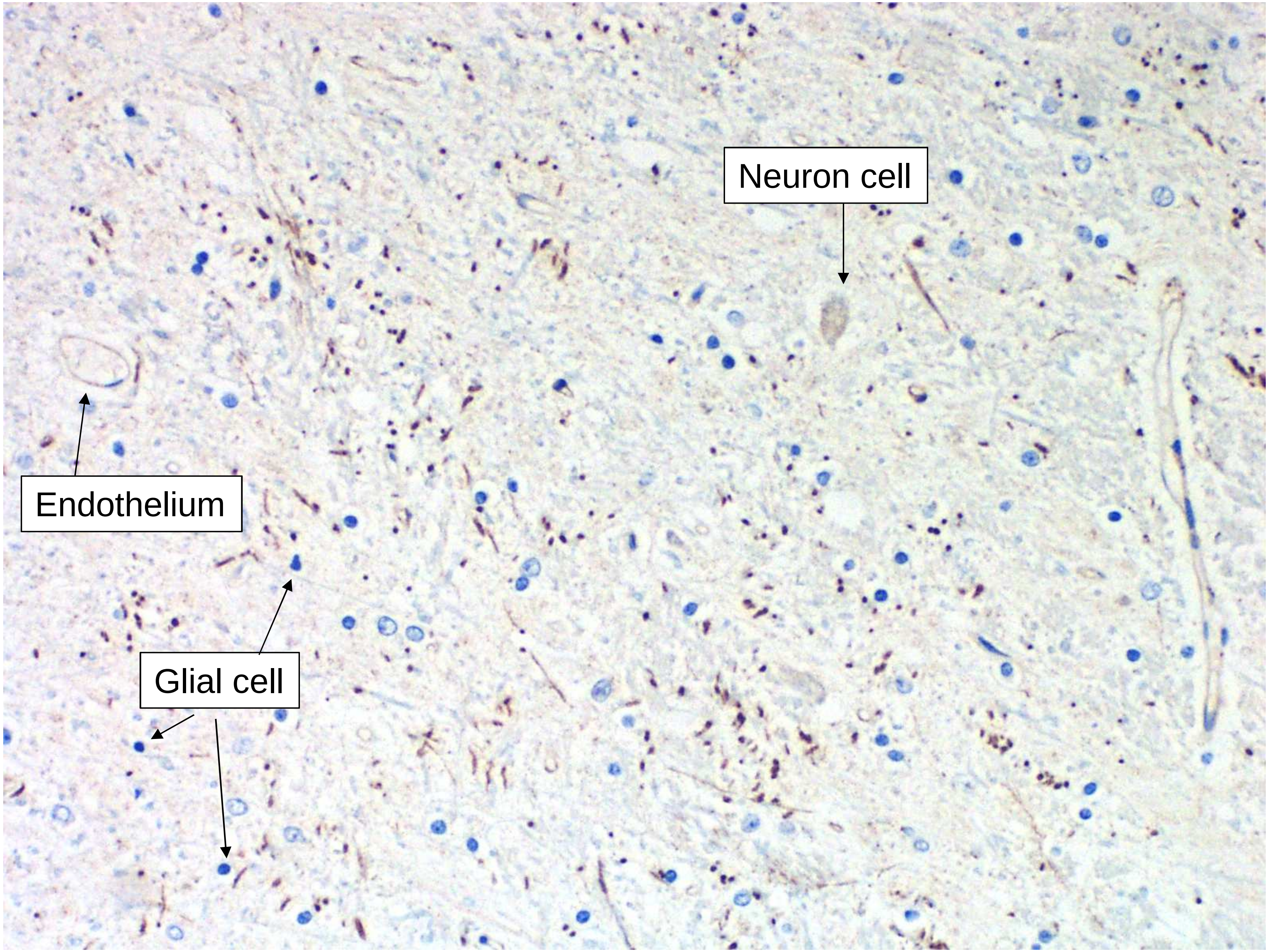

Globus pallidus: Granular positive staining of neuropil. Neuronal cells, glial cells and endothelium are negative (20X).
